# Isoform-specific patterns of tau burden and neuronal degeneration in *MAPT*-associated frontotemporal lobar degeneration

**DOI:** 10.1007/s00401-022-02487-4

**Published:** 2022-09-06

**Authors:** Lucia A. A. Giannini, Daniel T. Ohm, Annemieke J. M. Rozemuller, Laynie Dratch, EunRan Suh, Vivianna M. van Deerlin, John Q. Trojanowski, Edward B. Lee, John C. van Swieten, Murray Grossman, Harro Seelaar, David J. Irwin, Netherlands Brain Bank

**Affiliations:** 1 Alzheimer Center, Department of Neurology, Erasmus University Medical Center, Doctor Molewaterplein 40, 3015 GD Rotterdam, The Netherlands; 2 Digital Neuropathology Laboratory, Department of Neurology, Perelman School of Medicine, University of Pennsylvania, Philadelphia, PA 19104 USA; 3 Frontotemporal Degeneration Center (FTDC), University of Pennsylvania Perelman School of Medicine, Hospital of the University of Pennsylvania, 3600 Spruce Street, Philadelphia, PA 19104 USA; 4 Department of Pathology, Amsterdam Neuroscience, Amsterdam University Medical Center, Location VUmc, Amsterdam, The Netherlands; 5 Center for Neurodegenerative Disease Research, Department of Pathology and Laboratory Medicine, Perelman School of Medicine, University of Pennsylvania, Philadelphia, PA 19104 USA; 6 Translational Neuropathology Research Laboratory, Department of Pathology and Laboratory Medicine, Perelman School of Medicine, University of Pennsylvania, Philadelphia, PA 19104 USA

**Keywords:** Frontotemporal lobar degeneration, Genetic tauopathies, Tau pathology, Neuronal degeneration

## Abstract

Frontotemporal lobar degeneration with *MAPT* pathogenic variants (FTLD-MAPT) has heterogeneous tau pathological inclusions postmortem, consisting of three-repeat (3R) or four-repeat (4R) tau isoforms, or a combination (3R + 4R). Here, we studied grey matter tau burden, its relation to neuronal degeneration, and regional patterns of pathology in different isoform groups of FTLD-MAPT. We included 38 FTLD-MAPT autopsy cases with 10 different *MAPT* pathogenic variants, grouped based on predominant tau isoform(s). In up to eleven regions (ten cortical and one striatal), we quantified grey matter tau burden using digital histopathological analysis and assigned semi-quantitative ratings for neuronal degeneration (i.e. 0–4) and separate burden of glial and neuronal tau inclusions (i.e. 0–3). We used mixed modelling to compare pathology measures (1) across the entire cohort and (2) within isoform groups. In the total cohort, tau burden and neuronal degeneration were positively associated and most severe in the anterior temporal, anterior cingulate and transentorhinal cortices. Isoform groups showed distinctive features of tau burden and neuronal degeneration. Across all regions, the 3R isoform group had lower tau burden compared to the 4R group (*p* = 0.008), while at the same time showing more severe neuronal degeneration than the 4R group (*p* = 0.002). The 3R + 4R group had an intermediate profile with relatively high tau burden along with relatively severe neuronal degeneration. Neuronal tau inclusions were most frequent in the 4R group (*p* < 0.001 vs. 3R), while cortical glial tau inclusions were most frequent in the 3R + 4R and 4R groups (*p* ≤ 0.009 vs. 3R). Regionally, neuronal degeneration was consistently most severe in the anterior temporal cortex within each isoform group. In contrast, the regions with the highest tau burden differed in isoform groups (3R: striatum; 3R + 4R: striatum, inferior parietal lobule, middle frontal cortex, anterior cingulate cortex; 4R: transentorhinal cortex, anterior temporal cortex, fusiform gyrus). We conclude that FTLD-MAPT isoform groups show distinctive features of overall neuronal degeneration and regional tau burden, but all share pronounced anterior temporal neuronal degeneration. These data suggest that distinct isoform-related mechanisms of genetic tauopathies, with slightly divergent tau distribution, may share similar regional vulnerability to neurodegeneration within the frontotemporal paralimbic networks.

**Supplementary Information:**

The online version contains supplementary material available at 10.1007/s00401-022-02487-4.

## Introduction

The neuropathological spectrum underlying frontotemporal dementia (FTD) is highly heterogeneous. While approximately half of the cases are caused by frontotemporal lobar degeneration (FTLD) pathology with inclusions of the protein TDP-43 (FTLD-TDP), another substantial portion (40%) of the cases are caused by FTLD pathology with inclusions of the protein tau (FTLD-Tau) [50]. These pathologies can present in familial forms and have an underlying genetic aetiology in approximately one-third of the cases [30]. In particular, familial forms of FTLD-Tau are due to pathogenic variants in the microtubule-associated protein tau (*MAPT*) gene (FTLD-MAPT) [43].

Tau is a microtubule-binding protein, highly expressed in neurons and to a lesser extent in glial cells, which promotes microtubule assembly and stability, essential for neuronal integrity [75]. *MAPT* was the first gene to be associated with FTLD more than two decades ago [33, 55, 65]; to date, more than 60 pathogenic variants in the *MAPT* gene have been reported [30]. The tau-encoding *MAPT* gene, located on chromosome 17q21, gives rise to six different tau isoforms in the adult brain due to alternative splicing of exons 2, 3 and 10. In particular, alternative splicing of exon 10 produces tau isoforms with either three-repeat (3R) or four-repeat (4R) domains.

FTLD-MAPT pathology is associated with highly heterogeneous tau immunoreactivity postmortem, with a wide range of neuropathological inclusions in neurons and glia, and differential involvement of 3R or 4R tau isoforms [22, 25, 43]. These pathologic characteristics differ in each variant, and sometimes even within the same families; the cause of this variation is unclear [25]. While few studies have attempted to comprehensively review the spectrum of pathology associated with these variants [22, 25], no study to date has objectively quantified tau burden, or directly compared specific isoform subtypes of FTLD-MAPT. Further, while these variants are known to cause severe frontal and temporal lobe atrophy antemortem [78, 81]*,* the relationship between microscopic neurodegeneration and tau burden postmortem has scarcely been studied. Detailed study of this relationship may help to understand the patterns of gross cortical atrophy in living patients.

Here, we measured tau burden and its relation to neuronal degeneration in the largest cohort of FTLD-MAPT described to date, including ten different variants from 38 patients. We found that FTLD-MAPT variants with 3R tau are associated with relatively low tau burden, but very severe neuronal degeneration, in contrast to 4R variants showing relatively high tau burden (both neuronal and glial) and less severe neuronal degeneration, while in 3R + 4R variants both tau burden (especially glial tau) and neuronal degeneration were relatively high. Moreover, we found somewhat divergent regional patterns of tau burden in different isoform groups, but a shared vulnerability of the anterior temporal lobe to neuronal degeneration across all groups. These novel data help elucidate patterns of regional tau spread and associated neuronal degeneration, and shed light on pathologic heterogeneity in tauopathies.

## Materials and methods

### Patients

Patients were selected from the Netherlands Brain Bank and the Center for Neurodegenerative Disease Research at the University of Pennsylvania Perelman School of Medicine. Of autopsied subjects with confirmed FTLD-MAPT pathogenic variants that came to autopsy from 1993 until 2020 (*N* = 40), we excluded two cases with a P301L variant because of insufficient tissue available for analysis (i.e. < 3 regions available). In total, we included 38 patients. Of these, 26 were diagnosed and/or treated by a neurologist at the Erasmus University Medical Center, Rotterdam, the Netherlands and autopsied at the Netherlands Brain Bank (i.e. Erasmus cohort), while 12 patients were autopsied at the University of Pennsylvania (i.e. Penn cohort). Patients of the Penn cohort were clinically evaluated at the Penn Frontotemporal Degeneration Center with the exception of five who were referred from external clinics, where hemispheres were harvested locally and fixed brain tissue was shipped to Penn for neuropathological examination, using otherwise standardized approaches comparable to internal Penn cases.

### Clinical data

Available clinical data were collected from clinical charts from the Erasmus University Medical Center or Penn Frontotemporal Degeneration Center in a standardized manner by an experienced investigator (DJI, LAAG), as described [27, 35, 38]. Briefly, we obtained information about clinical features of language, behaviour, motor and other cognitive domains. The cohort was characterized based on baseline clinical data (within 3 years of disease onset), gathered from the clinical history and bedside neurologic examinations.

### Neuropathological diagnosis

Brain autopsy was carried out according to the Legal and Ethical Code of Conduct of the Netherlands Brain Bank or of the University of Pennsylvania. At the Netherlands Brain Bank, tissue collected at autopsy from one hemisphere was fixed in 10% neutral buffered formalin for 4 weeks and subsequently embedded in paraffin blocks. At the University of Pennsylvania, fresh samples from standard regions were obtained and fixed overnight in either 70% ethanol with 150 mM NaCl or 10% neutral buffered formalin and embedded in paraffin blocks. Paraffin-embedded tissue was cut into 6 µm sections for immunohistochemical staining for tau, Aβ, TDP-43 and alpha-synuclein with well-characterized antibodies [70]. Neuropathological diagnosis was performed by an expert neuropathologist at the Netherlands Brain Bank (AJMR) or at the University of Pennsylvania (EBL, JQT) using established criteria [51, 54]. All patients had a neuropathological diagnosis of FTLD-Tau. Further histological characterization of the cohort was performed by two researchers experienced in neuropathological assessments (DJI, LAAG), achieving consensus on the occurrence and frequency of tau inclusions in each case using published morphological criteria [42–44].

### Genetic analysis

All cases were screened and tested positively for a pathogenic genetic variant in the *MAPT* gene, with the exception of one case who did not undergo genetic testing but came from a family carrying the L315R variant and had clinical and pathological features typical of this variant. An overview of genetic information, specific *MAPT* variants and familial relationships between the cases, when known, can be found in Supplementary Table 1, online resource.

### Brain sampling

We examined ten cortical regions that have been implicated in FTLD-MAPT, including the anterior cingulate gyrus (ACG, Brodmann area [BA] 24), anterior temporal cortex (ATC, BA 38/20), entorhinal cortex (EC, BA 28), frontobasal (orbitofrontal) cortex (FBC, BA 10/11), fusiform gyrus (FG, BA 37), inferior parietal lobule (IPL, BA 39), middle frontal cortex (MFC, BA 46), superior temporal gyrus (STG, BA 22), transentorhinal cortex (TEC, BA 35) and visual cortex (VC, BA 17). The visual cortex served as an internal control region with limited pathology. Additionally, we included the striatum (STRI) as additional subcortical region implicated in FTLD-MAPT [25, 65]. Minor differences in sampling were present across the two centres; the FBC comprises the frontal pole (BA 10) of the Erasmus cohort and the orbitofrontal cortex (BA 11) of the Penn cohort; the ATC comprises the temporal pole (BA 38) of the Erasmus cohort and the anterior-inferior temporal cortex (BA 20) of the Penn cohort. The STG was the only region that was exclusively sampled at one centre (in the Penn cohort), while all other regions included data from both the Erasmus and the Penn cohort. Tissue was sampled from one hemisphere in each case; this was the right hemisphere in almost all cases of the Erasmus cohort (right: *N* = 25; left: *N* = 1), while in the Penn cohort the hemisphere was selected for sampling in a random fashion (right hemisphere *N* = 3; left hemisphere *N* = 7). For two external cases of the Penn cohort, the sampled hemisphere was not recorded (*N* = 2). Two more recent cases of the Penn cohort had additional bilateral sampling in a subset of regions (i.e. ATC, FBC and STG in one case with P301L variant; ACG, ATC, FBC, STG, STRI and TEC in one case with IVS10 + 16 variant).

### Immunohistochemistry

We performed immunohistochemistry at the Penn Digital Neuropathology laboratory using AT8 antibody for phosphorylated tau (pre-)inclusions and NeuN for neurons using an identical protocol that was optimized for both cohorts. AT8 immunohistochemistry was performed using anti-AT8 (#MN1020, Invitrogen; dilution 1:1000) as primary antibody. NeuN immunohistochemistry was performed using anti-NeuN (#MAB377, Millipore; dilution 1:1000) and the signal was amplified using the Vector ABC Kit (#PK-6100). In a subset (3 legacy cases of the Erasmus cohort from 1993 to 1996, 2 cases of the Penn cohort processed externally; total *N* = 5), the quality of NeuN immunohistochemistry was suboptimal in all stained tissue sections in two repeated staining attempts, likely due to slightly different perimortem processing methods. In this subset, neuronal degeneration was evaluated based on haematoxylin staining rather than NeuN staining, as validated and described in detail below.

### Digital pathological analysis of tau burden

We digitally quantified burden of AT8-positive tau pathology as the percentage of area occupied (%AO) by tau-positive pixels in grey matter (GM) regions of interest (ROI) as described [27, 28, 36, 38] blinded to *MAPT* genetic variant. In cortical regions, if tissue areas were variably affected, we focused our sampling on the tissue area with the most severe neurodegeneration. Cortical GM ROIs were obtained using a transect belt sampling method as the longest stretch of parallel cortex to avoid bias from overrepresentation of a subset of cortical layers (Supplementary Fig. 1a, online resource) [1, 2]. In the striatum, we randomly placed four GM ROIs of 1 mm^2^ each, distributed across the striatal tissue (Supplementary Fig. 1b, online resource); we performed this sampling with the DAB channel off on QuPath, i.e. blinded to the severity of tau pathology. Further, to reduce sampling bias, the mean from a random sample of 175 × 175 µm tiles occupying 30% of each GM ROI was used to generate the %AO measurement as described previously [36, 38]. The %AO in each ROI was measured using empirically derived RGB detection algorithms and optical density (OD) thresholds tested and optimized based on visual inspection of a set of four to five slides from the same staining batch as described [36].

As digital pathology has been shown to be sensitive to staining batch effects [28], several previously validated measures were applied to minimize batch-related bias. Pathology data were stained in three batches for each cohort. Customized empirical detection algorithms were generated for each batch, and OD values were adjusted to minimize differences between the two batches using a set of slides stained in duplicate (i.e. measurements from 4 to 8 slides). Mean bias was assessed using Bland–Altman plots. Final detection algorithms and OD values (Supplementary Table 2, online resource) enabled obtaining negligible mean bias approaching zero (Supplementary Fig. 2, online resource). Interindividual variability was accounted for statistically using mixed modelling (see “Statistical analysis”). To account for potential bias due to batch effects in the two different cohorts (i.e. Erasmus and Penn cohorts), we reran all main analyses covarying for cohort and found no significant confounding effect (data not shown). Finally, digital pathology scores of tau burden were validated by comparison to traditional ordinal scores (i.e. 0–3), obtained blinded to quantitative pathology measurements (Supplementary Fig. 3, online resource) as previously described [36]. Additionally, we assigned ordinal scores for both neuronal and glial tau pathology (i.e. 0–3) in each sampled GM region to assess the relative severity of neuronal/glial tau inclusions.

Our total dataset consisted of 332 GM %AO measurements from 38 patients, acquired from 267 stained tissue slides. Missing data and damaged tissue were excluded from the analyses. We provide an overview of all available %AO measurements per region, *MAPT* variant and isoform group in Supplementary Table 3, online resource.

### Neuronal degeneration phase classification

Cortical tissue sections stained with NeuN were assessed systematically using an ordinal scale for neuronal degeneration comprising several interrelated parameters, i.e. cortical lamination, neuronal density, vacuolation and intraneuronal NeuN reactivity (Supplementary Fig. 4, online resource). This neuronal degeneration phase (NDP) classification was developed based on pattern recognition by a single rater (LAAG) and subsequently discussed and optimized in a consensus meeting including researchers experienced in neuropathology (DJI, DTO, HS, LAAG). Briefly, phase 0 corresponded to a normal cortex, while phases 1–4 corresponded to mild, moderate, severe and very severe neuronal degeneration, respectively. NDP scores were assigned blinded to *MAPT* genetic variant. Interrater agreement analyses in a subset of slides (*N* = 81, 25% of all assigned scores) showed optimal agreement (kappa = 0.82, *p* < 0.001) across two raters (DJI, LAAG) after joint training through discussion and scoring of a few example slides. A similar scale was adapted and optimized for the striatum, focusing on parameters of neuronal density, vacuolation and intraneuronal NeuN reactivity (Supplementary Fig. 5, online resource). Striatal samples (*N* = 29) were scored by two independent raters (DJI, LAAG) using this scale, minor discrepancies of one point in a subset of cases were discussed and consensus was achieved between the two raters. To complement scores in sampled regions where NeuN-stained tissue was missing (*N* = 51, 15%), NDPs were assigned by the same rater (LAAG) based on haematoxylin counterstain in AT8-stained slides (with digital suppression of DAB signal in QuPath for unbiased scoring), after adequate training in the whole cohort. Optimal agreement was found between NeuN-based NDPs and haematoxylin-based NDPs (kappa = 0.84, *p* < 0.001; Supplementary Fig. 6a, online resource). NDPs were further validated by comparison to conventional ordinal ratings of neuronal loss (Supplementary Fig. 6b, online resource). One occipital section from a P301L case was excluded from neuronal degeneration assessment due to a local infarction.

### Statistical analysis

Continuous demographic and clinical data were compared between the groups using analysis of variance (ANOVA), and, where applicable, post hoc comparisons were performed using pairwise independent samples *t* tests with Bonferroni correction for multiple comparisons. Categorical variables were compared between the groups using the Chi-squared test. Pathology data analysis was performed using mixed modelling, either linear or ordinal as appropriate, to account for multiple measurements from the same individual and for missing data. GM tau burden as a continuous measure was tested using linear mixed modelling after natural log transformation to obtain a normal distribution. In case model predictors of linear mixed-effects models (LME) included multiple categories (> 2), we also performed a type III ANOVA with Satterthwaite's method to assess the overall effect of these predictors, and, where applicable, planned post hoc pairwise comparisons using LME-derived least-square means with Tukey correction for multiple comparisons. Neuronal degeneration phase (i.e. NDP) scores and neuronal/glial tau ordinal scores as ordinal data were tested using ordinal mixed regression (i.e. cumulative link-mixed model). For these models, we tested the overall effect of model predictors using the likelihood ratio test (LRT) to compare the full model to one nested model with the same variables, but excluding the main predictor. Details of each model can be found in the Supplementary Methods, online resource. Finally, normalized GM tau burden in each of the five most affected regions was compared between isoform groups using ANCOVA, covarying for NDP, followed by planned post hoc analyses with Bonferroni correction for multiple comparisons. For these direct between-isoform group comparisons, natural log-transformed GM tau burden was further normalized within *MAPT* variant groups using min–max normalization to account for morphological differences of tau inclusions in the different *MAPT* variants. All analyses were performed using R statistical software 4.0.3.

## Results

### Patients

Our cohort of 38 FTLD-MAPT autopsy cases consisted of 7 cases with 3R-predominant variants (6 G272V [4], 1 dK280 [74]); 9 cases with 3R + 4R-mixed variants (2 L266V [32, 41], 2 L315R [73], 3 R406W [11, 49], 1 G389R [24], 1 S320F [57]); 22 cases with 4R-predominant variants (17 P301L [11, 22, 53], 4 IVS10 + 16 [11, 22], 1 N279K [11, 12]). All except one patient (35/36 = 97.2%) presented with behavioural variant FTD (bvFTD). One patient (1/36 = 2.8%) with an IVS10 + 16 variant had a clinical diagnosis of progressive supranuclear palsy (PSP). Two patients could not be characterized according to current clinical criteria due to limited clinical data. Parkinsonian features were reported in 14/34 (41.2%) of cases either at baseline (2/34, 5.9%) or at follow-up (12/34, 35.3%; *N* = 4 missing data). Clinical features per case are presented in Supplementary Table 4, online resource.

Comparison of age at onset in the different isoform groups (3R vs. 3R + 4R vs. 4R) showed a relatively earlier age at onset in the 3R and 3R + 4R groups compared to the 4R group (trend of *p* = 0.071 for 3R; *p* = 0.037 for 3R + 4R; Bonferroni-corrected post hoc), while disease duration and age at death were similar between isoform groups (*p* > 0.05). Other demographic or autopsy-related variables did not differ between the groups (Table 1).

**Table 1:** Demographic, clinical and autopsy features of the cohort

Case	Mutation	Isoform	Gender	Phenotype	Age at onset (y)*	Age at death (y)	Disease dur (y)	PMI (hrs)	Brain weight (gr)	Co-pathology
1	G272V	3R	F	bvFTD	45	54	9	5.7	791	
2	G272V	3R	F	bvFTD	47	67	20	4.2	757	
3	G272V	3R	F	bvFTD	47	54	7	7.5	1027	
4	G272V	3R	M	bvFTD	41	49	8	5.2	1188	Amyloid (A1, C0)
5	G272V	3R	M	bvFTD	42	51	9	4.4	962	
6	G272V	3R	M	bvFTD	42	49	7	5.1	1105	
7	dK280	3R	F	bvFTD	52	63	11	n/a	n/a	
Summary 3R			4 F, 3 M	7 bvFTD	45.1 ± 3.9^&^	55.3 ± 7	10.1 ± 4.6	5.3 ± 1.2	971.7 ± 171.1	
8	L266V	3R + 4R	F	bvFTD	31	34	3	n/a	840	
9	L266V	3R + 4R	F	bvFTD	24	31	7	12	870	
10	L315R	3R + 4R	F	bvFTD	54	63	9	4.2	758	
11	L315R	3R + 4R	M	bvFTD	52	57	5	7.6	1111	
12	R406W	3R + 4R	M	bvFTD	50	70	20	5.4	1066	
13	R406W	3R + 4R	F	bvFTD	58	71	13	5.8	849	Amyloid (A2, C2)
14	R406W	3R + 4R	F	bvFTD	58	75	17	7.4	946	
15	G389R	3R + 4R	F	bvFTD	40	43	3	12	1074	
16	S320F	3R + 4R	M	bvFTD	38	53	15	4.6	1160	
Summary 3R + 4R			6 F, 3 M	9 bvFTD	45.0 ± 12.3^#^	55.2 ± 16.3	10.2 ± 6.3	7.4 ± 3.1	963.8 ± 142.6	
17	P301L	4R	F	bvFTD	54	66	12	5.7	701	
18	P301L	4R	M	bvFTD	45	52	7	6.7	997	
19^¥^	P301L	4R	F	bvFTD	56	76	20	6.3	886	
20	P301L	4R	M	bvFTD	58	66	8	5	1011	
21	P301L	4R	M	bvFTD	39	46	7	5.6	1065	
22	P301L	4R	F	bvFTD	56	66	10	6.7	707	
23	P301L	4R	M	bvFTD	49	52	3	11.5	1115	
24	P301L	4R	F	FTLD-NOS	51	71	20	n/a	894	
25	P301L	4R	F	bvFTD	56	65	9	6	970	
26	P301L	4R	M	bvFTD	51	60	9	5.4	887	
27	P301L	4R	F	bvFTD	50	64	14	7.5	652	
28	P301L	4R	M	bvFTD	52	64	12	6.4	1029	
29^¥^	P301L	4R	M	bvFTD	53	55	2	4.4	1100	
30	P301L	4R	F	bvFTD	53	64	11	n/a	900	
31	P301L	4R	M	bvFTD	57	68	11	n/a	1120	Amyloid (A1, C2)
32	P301L	4R	M	bvFTD	53	57	4	5.2	1197	
33	P301L	4R	M	bvFTD	57	65	8	5.5	1002	
34	IVS10 + 16	4R	M	PSP	46	48	2	12	1580	
35	IVS10 + 16	4R	F	bvFTD	55	62	7	6	1113	Amyloid (A1, C0)
36	IVS10 + 16	4R	M	bvFTD	60	68	8	11	1382	Amyloid (A1, C0)
37	IVS10 + 16	4R	F	bvFTD	54	64	10	10	1189	
38	N279K	4R	F	FTLD-NOS	n/a	49	n/a	5	945	
Summary 4R			10 F, 12 M	19 bvFTD, 2 FTLD-NOS, 1 PSP	52.6 ± 4.9	61.3 ± 7.9	9.2 ± 4.8	6.9 ± 2.4	1020.1 ± 212.8	

*bvFTD * behavioural variant frontotemporal dementia, *dur*  duration, *F*  female, *FTLD-NOS*  frontotemporal lobar degeneration not otherwise specified, *M*  male, *n/a* not available, *PSP* progressive supranuclear palsy

^¥^Lateralized atrophy at gross macroscopic examination was reported in only two cases: case 19 had left-lateralized atrophy particularly in the temporal lobe; case 29 had right-lateralized atrophy

^*^Isoform groups differed in age at onset (*F* = 5.0, *df* = 2,34, *p* = 0.013; ANOVA)

^&^*p* = 0.071 compared to the 4R group (Bonferroni-corrected post hoc tests)

^#^*p* = 0.037 compared to the 4R group (Bonferroni-corrected post hoc tests)

**Fig. 1:**
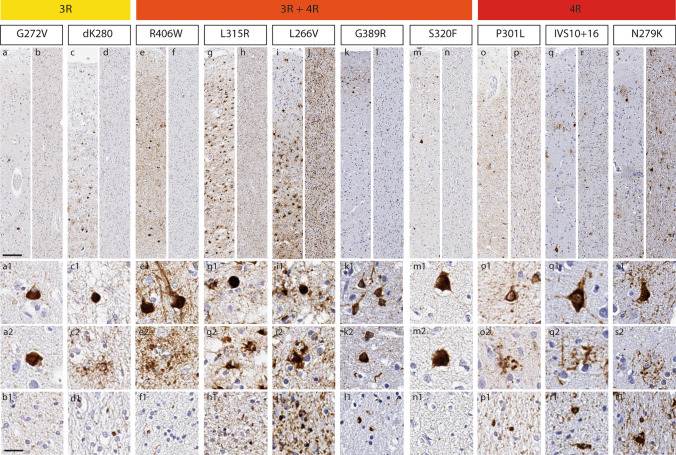
Morphological features of tau inclusions in different *MAPT* variants**.** For each *MAPT* variant in different isoform groups, we show typical inclusions. 3R variants included the G272V (**a**–**b**) and the dK280 (**c**–**d**) variants. In grey matter, the G272V variant had predominantly neuronal pathology appearing as diffuse neuronal cytoplasmic inclusions (DNCI, a1), neurofibrillary tangle-like inclusions (NFTLI), or Pick body-like inclusions (PBLI; a2), with variable amounts of grain-like threads; glial pathology was negligible and in white matter there were no or rare intracellular inclusions (b1). The dK280 variant showed frequent Pick body-like inclusions (PBLI; c1), DNCIs and a moderate amount of ramified astrocytes (RA; c2) in grey matter, along with other astrocytic morphologies; in white matter, few small globular oligodendrocytic inclusions (GOI) were present (d1). 3R + 4R variants included the R406W (**e**–**f**), L315R (**g**–**h**), L266V (**i**–**j**), G389R (**k**–**l**) and S320F (**m**–**n**). In grey matter, the R406W variant was characterized by abundant Alzheimer-like NFTLIs (e1), neuritic threads (NT) and various astrocytic inclusions, including frequent astrocytic plaques (AP; e2); in white matter, small GOIs and threads were found (f1). The L266V and L315R variants had a similar morphological profile showing frequent PBLIs (g1; i1), ramified and punctate astrocytes (g2; i2) in grey matter, and diffuse threads (DT), small coiled bodies (CB) and small globular oligodendrocytic inclusions (GOI) in white matter (h1; j1). The G389R and S320F variants showed moderate–severe amounts of neuronal pathology, including NFTLI (k1, m1), DNCIs (k2) or PBLIs (m2) in grey matter, and few short threads in both grey and white matter (l1; n1); in both variants, intracellular glial pathology was negligible. 4R variants included the P301L (o–p), IVS10 + 16 (q–r) and N279K (s–t) variants. The P301L variant was characterized by moderate to severe amounts of neuronal pathology, including perinuclear rings (PNR, o1), DNCIs and NFTLIs, and many tau-positive diffuse grey matter threads; there was mild-moderate astrocytic pathology in grey matter, appearing often as globular astrocytic inclusions (GAIs; o2); white matter pathology included threads, variable amounts of small GOIs (p1) as well as CBs. The IVS10 + 16 and N279K variants presented similar pathological morphologies including DNCIs or NFTLIs (q1; s1), APs (q2; s2), threads as well as CBs in grey matter; in both variants, white matter pathology was abundant, including large CBs (r1; t1), GOIs and threads

The morphologies of neuropathological inclusions in different variants, largely concordant with previous reports [3, 4, 12, 22–24, 68, 72–74], are illustrated in Fig. 1. Additionally, we report predominant inclusions and their relative severity in each case in Table 2, demonstrating heterogeneity between and within specific variants. Most commonly, *MAPT* patients in the cohort had mixed and atypical histopathological features (i.e. unclassifiable tauopathies). The subset that more closely resembled morphological phenotypes of sporadic tauopathies included the L226V and L315R variants, appearing similar to sporadic Pick’s disease (PiD). Notably, a subset of P301L patients (cases 19, 24–26, 30–33) had mild to moderate globular tau inclusions in astrocytes and oligodendrocytes resembling sporadic globular glial tauopathy (GGT), and one patient with the IVS10 + 16 variant (case 36) had prominent white matter tau and astrocytic plaques that resembled sporadic corticobasal degeneration (CBD), while other IVS10 + 16 cases had more mixed glial tau morphologies (Table 2).

**Table 2:** Case-by-case neuropathological characterization of the cohort with tau inclusions’ morphologies and their severity

			Neuronal pathology					Astrocytic pathology								Oligodendrocytic pathology			Neuropil		
Case	Mutation	Isoform	TNT	DNCI	PBLI	NFTLI	PNR	TAT	TA	AP	RA	GAI	PA	TSA^a^	GFA	TOT	CB	GOI	GMG^b^	GMT	WMT
1	G272V	3R	+	±	+	+	-	±	-	-	-	-	-	±	-	-	-	-	±	±	-
2	G272V	3R	+	+	+	+	-	±	±	-	-	-	-	-	-	-	-	-	±	+	±
3	G272V	3R	+	+	+	+	-	±	±	-	-	-	-	-	-	-	-	-	+ +	+ +	-
4	G272V	3R	+ +	+	±	+ +	-	±	±	-	-	±	-	-	-	-	-	-	+	+ +	-
5	G272V	3R	+ +	+	±	+ +	-	±	±	-	-	-	-	-	-	-	-	-	+ +	+	±
6	G272V	3R	+ +	±	+	+ +	-	±	±	-	-	±	-	-	--	-	-	-	+ +	+	-
7	dK280	3R	+ +	±	+ +	+ +	-	+ +	+ +	+	+ +	+	+	+	±	+	+	+	-	+	+
8	L266V	3R + 4R	+ + +	+	+ + +	+	±	+ + +	+	-	+ + +	+	+ + +	+	±	+ + +	+ + +	-	±	+ +	+ + +
9	L266V	3R + 4R	+ + +	+ + +	+ + +	+	±	+ + +	+	+	+ + +	-	+ + +	+	±	+ +	+ +	+ +	-	+ + +	+ +
10	L315R	3R + 4R	+ +	±	+ +	+	-	+ + +	+ + +	-	+ + +	-	+ +	+	±	+	-	+	-	+	±
11	L315R	3R + 4R	+ + +	+	+ + +	+	-	+ + +	±	-	+ + +	-	+ +	+	-	+ + +	-	+ + +	-	+ +	+ +
12	R406W	3R + 4R	+ + +	+ +	+ +	+ + +	+ +	+ + +	+ + +	+	+ +	+ +	-	+	±	+	+	+	-	+ + +	+
13	R406W	3R + 4R	+ +	+	+	+ +	-	+ +	+	+ +	-	+	-	±	+ +	±	±	-	-	+ + +	±
14	R406W	3R + 4R	+ +	+	+ +	+ +	-	+ + +	+	+ + +	±	+ +	-	±	+	+ +	+ +	+ +	-	+ +	+ +
15	G389R	3R + 4R	+ +	+ +	-	+ +	+ +	-	-	-	-	-	-	-	-	+ +	+ +	+	±	+ +	+
16	S320F	3R + 4R	+ +	±	+ +	+ +	-	±	±	-	-	±	-	-	-	-	-	-	+	±	-
17	P301L	4R	+ + +	+ + +	±	+ +	+ +	±	±	-	-	±	-	-	±	+	+	+	** + **	+ + +	+
18	P301L	4R	+ + +	+ + +	+	+	+ + +	±	±	-	-	±	-	-	-	±	±	±	±	+ + +	+
19	P301L	4R	+ + +	+ + +	-	+ + +	+ + +	+ +	+	+ +	±	+	-	-	+ +	+	±	+	-	+ + +	+
20	P301L	4R	+ + +	+ +	-	+ + +	+ + +	±	±	±	-	±	-	-	±	±	-	±	-	+ + +	-
21	P301L	4R	+ +	+	±	+	+ +	±	±	-	-	-	-	-	-	±	±	±	±	+	±
22	P301L	4R	+ +	+	±	+	+ +	±	±	-	-	±	-	±	-	+	±	+	-	+	±
23	P301L	4R	+ + +	+ +	-	+ +	+ + +	±	±	-	-	-	-	-	-	±	-	±	±	+ +	-
24	P301L	4R	+ + +	+ + +	-	+	+ + +	+ +	+ +	+ +	-	+	-	+	+ +	+	+	±	±	+ + +	+
25	P301L	4R	+ + +	+ + +	-	+	+ + +	+ +	±	+ +	-	+	-	-	+ +	+	+	+	±	+ + +	+ +
26	P301L	4R	+ + +	+	-	+	+ + +	+	±	-	-	+	-	-	±	+	-	+	+ +	+ +	+
27	P301L	4R	+ + +	±	-	+	+ + +	±	-	±	-	-	-	-	-	±	±	-	-	+ + +	-
28	P301L	4R	+ + +	±	-	+	+ + +	±	±	-	-	±	-	-	-	±	-	±	-	+ + +	-
29	P301L	4R	+ + +	+	-	+	+ + +	±	-	-	-	±	-	-	-	+	+	+	-	+ + +	+
30	P301L	4R	+ + +	+ + +	±	+	+ + +	+ + +	+	±	+	+ + +	±	+	+ +	+ +	+	+ +	±	+ +	+ +
31	P301L	4R	+ + +	+ + +	-	+	+ +	+	±	+	±	+	-	-	+	+	+	+	±	+ +	+
32	P301L	4R	+ + +	+	-	+	+ + +	+	±	±	-	+	-	-	+	+	-	+	+	+ + +	+
33	P301L	4R	+ + +	+ +	-	+	+ + +	+	-	+	-	+	-	-	±	+	+	+	-	+ + +	+
34	IVS10 + 16	4R	+	+	-	+	±	+	+	+	-	-	-	±	+ +	+ +	+	+ +	±	+	+ +
35	IVS10 + 16	4R	+ +	+ +	-	+ +	+	+	+	+	-	+	-	±	+	+ + +	+ +	+ + +	+ + +	+ +	+ + +
36	IVS10 + 16	4R	+	+	-	+	+	+ + +	+	+ + +	+	-	-	±	+ + +	+ +	+ +	+ +	±	+	+ +
37	IVS10 + 16	4R	+	+	-	+	±	+	+	±	±	+	-	-	±	+ +	+	+ +	+ +	+	+ + +
38	N279K	4R	+	±	-	+	-	+ +	±	+ +	-	±	-	-	+ +	+ + +	+	+ + +	±	+	+ + +

*AP* astrocytic plaques, *CB* coiled bodies, *DNCI* diffuse neuronal cytoplasmic inclusions, *GAI* globular astrocytic inclusions, *GFA* granular fuzzy astrocytes, *GMG* grey matter grains, *GMT* grey matter threads, *GOI* globular oligodendrocytic inclusions, *NFTLI* neurofibrillary tangle-like inclusions, *PA* punctate astrocytes, *PBLI* Pick body-like inclusions, *PNR* perinuclear rings, *RA* ramified astrocytes, *TA* tufted astrocytes, *TAT* total astrocytic tau, *TNT* total neuronal tau, *TOT* total oligodendrocytic tau, *TSA* thorn-shaped astrocytes, *WMT* white matter threads.

Presence and overall severity of specific tau morphological inclusions are described for each case (- absent, ± sparse, + mild, +  + moderate, +  +  + severe/abundant)

^a^Presence of (ARTAG-like) thorn-shaped astrocytes (TSA) was defined as astrocytic inclusions with this morphology located around blood vessels and/or at subpial surfaces

^b^Grey matter grains (GMG) were marked as sparse ( ±) if they were only present in the hippocampus, while mild, moderate and severe scores were assigned when other cortical regions were also involved (+ mild/occasionally present, +  + moderate/consistently present, +  +  + severe/consistently present and abundant)

### Tau burden and neuronal degeneration in all FTLD-MAPT patients

**Fig. 2:**
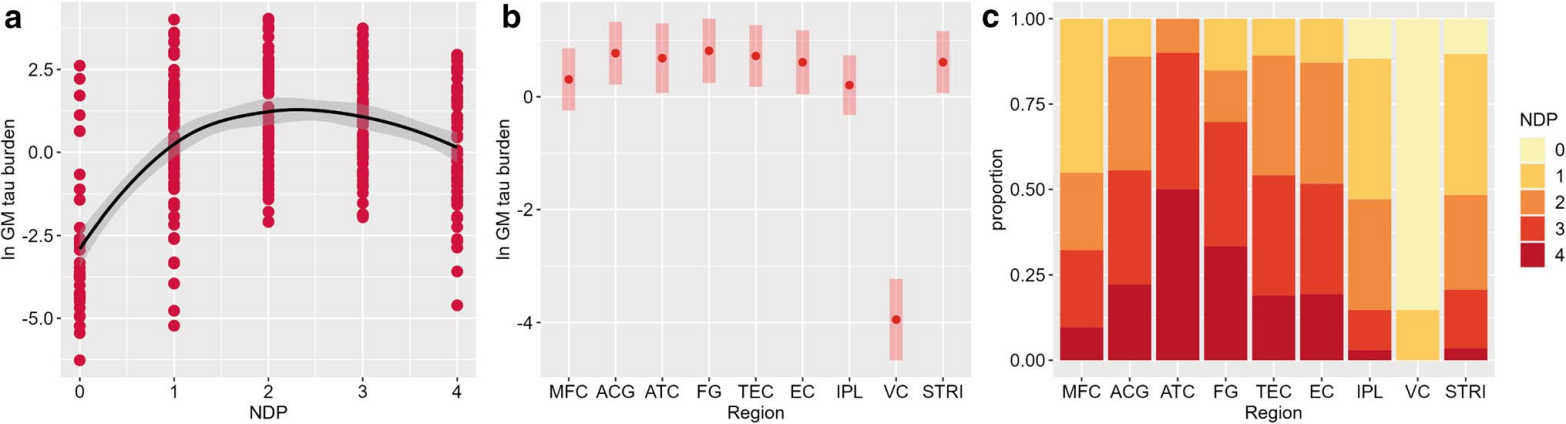
Grey matter tau burden and neuronal degeneration across the whole FTLD-MAPT cohort. Across FTLD-MAPT, neuronal degeneration and GM tau burden are positively associated, but GM tau burden shows a slight decline at most severe stages of neuronal degeneration (**a**). Regional analysis finds highest GM tau burden in the FG, ACG, TEC and ATC (**b**). These regions also show most severe neuronal degeneration, along with the EC (**c**). Legend: *ACG* anterior cingulate gyrus, *ATC* anterior temporal cortex, *EC* entorhinal cortex, *FG* fusiform gyrus, *GM* grey matter, *IPL* inferior parietal lobule, *MFC* middle frontal cortex, *NDP* neuronal degeneration phase, *STRI* striatum, *TEC* transentorhinal cortex, *VC* visual cortex. **a**/**b** ln GM tau burden indicates the percentage area occupied (%AO) by AT8-positive pixels in GM after natural log transformation. **b** Least-square means and 95% confidence intervals from the linear mixed-effect model are portrayed

Across the entire cohort, we found an association between increasing NDP score and GM tau burden (*F* = 7.0, *df* = 4,293, *p* = 0.001). Graphically, across the whole cohort, we observed a gradual increase in GM tau burden from NDP score 0 to 2 overall, followed by a slight decline in GM tau burden at NDP scores 3 and 4, suggesting loss of tau positivity at severe and very severe stages of neuronal degeneration (Fig. 2a).

Studying the regional distribution of GM tau burden, we found that the fusiform gyrus (FG; least-square mean = 0.82, SE = 0.29), anterior cingulate gyrus (ACG; least-square mean = 0.77, SE = 0.28), transentorhinal cortex (TEC; least-square mean = 0.72, SE = 0.28) and anterior temporal cortex (ATC; least-square mean = 0.68, SE = 0.31) were the regions with the highest tau burden (Fig. 2b). Concurrently, the ATC had the most severe neuronal degeneration (i.e. NDP score of 4 in 50.0% of all ATC assessments), followed by the FG (33.3%), ACG (22.2%), EC (19.4%) and TEC (18.9%) (LRT = 322.2, *df* = 8, *p* < 0.001; Fig. 2c).

### Tau burden and neuronal degeneration in isoform groups

**Fig. 3:**
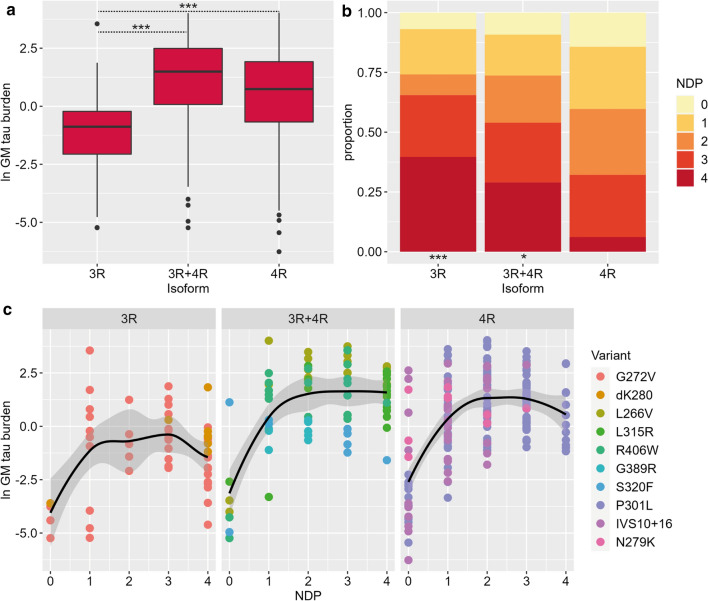
Grey matter tau burden and neuronal degeneration in different isoform groups. The 3R isoform group shows lower GM tau burden compared to the 3R + 4R and 4R groups (*p* ≤ 0.008; **a**), but greater frequency of most severe neuronal degeneration (NDP = 4), together with the 3R + 4R group, compared to the 4R group (*p* ≤ 0.04; **B**). The relation between neuronal degeneration and GM tau burden is relatively similar within each isoform group, but the slight decline in GM tau burden at most severe stages of neuronal degeneration is most prominent in the 3R group compared to the other groups (**c**). Legend: *GM* grey matter, *NDP* neuronal degeneration phase. In panels **a**/**c**: ln GM tau burden indicates the percentage area occupied (%AO) by AT8-positive pixels in GM after natural log transformation. ***p < 0.01. *p < 0.05

Comparing the overall severity of GM tau burden and neuronal degeneration phase (i.e. NDP) between different isoform groups, we found that isoform groups differed significantly in the severity of overall GM tau burden (*F* = 7.5, *df* = 2,36, *p* = 0.002; Fig. 3a). The 3R isoform group was associated with overall lower GM tau burden compared to the 3R + 4R (beta = − 2.17, SE = 0.58, *p* = 0.002) and 4R isoform groups (beta = -1.59, SE = 0.50, *p* = 0.008). Isoform groups also differed in the severity of neurodegeneration (LRT = 12.6, *df* = 2, *p* = 0.002). The 3R group, as well as the 3R + 4R group, had relatively high neuronal degeneration, which for both groups was greater than that in the 4R group (3R: log odds = 2.84, SE = 0.83, *p* = 0.002; 3R + 4R log odds = 1.84, SE = 0.74, p = 0.040; Fig. 3b).

Next, we looked at the relationship between NDP score and GM tau burden in each isoform group. A gradual increase in GM tau burden from NDP score of 0–2 overall, followed by a slight decline or plateau at NDP scores 3–4, was observed in all isoform groups. Notably, the 3R isoform group showed the most evident decline in GM tau burden at severe phases of neuronal degeneration (Fig. 3c). This trend of decreasing tau burden at severe NDP scores was especially evident in the 3R group when restricting the analysis to only cortical samples, excluding the striatum (Supplementary Fig. 7, online resource). Specific *MAPT* variants showed largely consistent findings with some variability in patterns of tau burden and neuronal degeneration, which may in part be due to the small sample size in some variants (Supplementary Fig. 8–9, online resource).

### Relative severity of cortical neuronal and glial pathology

**Fig. 4:**
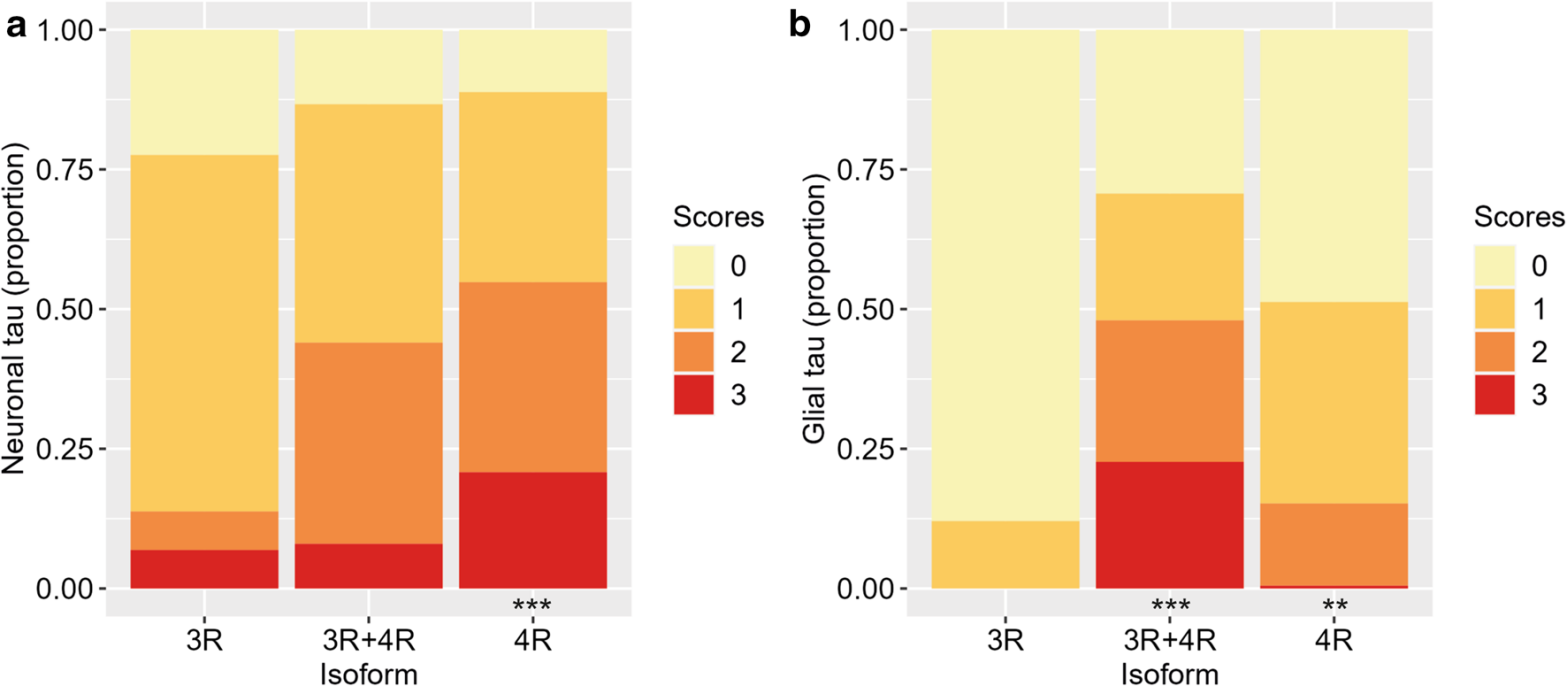
Relative severity of neuronal and glial tau pathology in different isoform groups. Isoform groups differ in the relative severity of neuronal (**a**) and glial (**b**) tau pathology (i.e. ordinal scores 0–3). The 4R isoform group has greatest severity of neuronal pathology, which is more severe than that in the 3R group (*p* < 0.001), while the 3R + 4R group has greatest severity of glial pathology, which is more severe than that in the 3R group (*p* < 0.001). Additionally, the 4R group has greater glial pathology than the 3R group (*p* = 0.009). ***p < 0.001 vs. 3R. **p < 0.01 vs. 3R

When looking at the relative severity of neuronal/glial pathology in each isoform group, we found that the 4R isoform group had most severe neuronal tau pathology, which was greater than that in the 3R group (log odds = 2.76, SE = 0.74, *p* < 0.001; Fig. 4a). As for glial pathology, the 3R + 4R group had the most severe glial pathology, which was significantly greater than that in the 3R group (log odds = 8.94, SE = 2.14, *p* < 0.001; Fig. 4b). Also, the 4R group showed greater glial pathology than the 3R group (log odds = 5.50, SE = 1.85, *p* = 0.009). Specific *MAPT* variants stood out as having the greatest neuronal pathology (4R: P301L; 3R + 4R: L266V, L315R; Supplementary Fig. 10a, online resource) or glial pathology (3R + 4R: L315R, L266V, R406W; 4R: IVS10 + 16; Supplementary Fig. 10b, online resource).

### Regional patterns of pathology in isoform groups

**Fig. 5:**
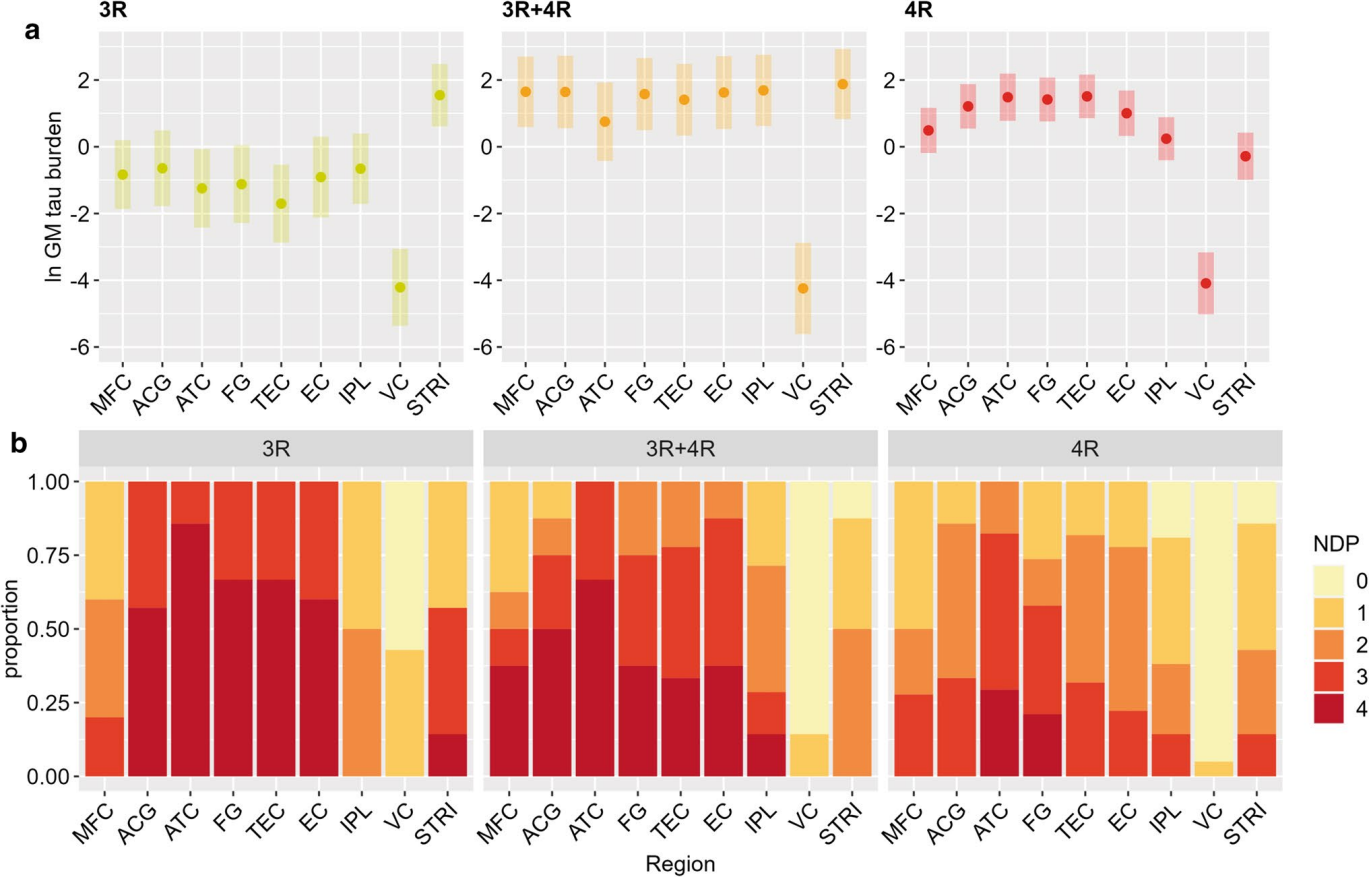
Regional distribution of grey matter tau burden and neuronal degeneration in different isoform groups. Regional analysis finds highest GM tau burden in the STRI in the 3R isoform group, in the STRI closely followed by other regions (IPL, MFC, ACG) in the 3R + 4R isoform group, and in the TEC, ATC and FG in the 4R isoform group (**a**), in part discordant with neuronal degeneration findings, highlighting most severe neuronal degeneration in the ATC regardless of isoform group (**b**). Legend: *ACG* anterior cingulate gyrus, *ATC* anterior temporal cortex, *EC* entorhinal cortex, *FG* fusiform gyrus, *GM* grey matter, *IPL* inferior parietal lobule, *MFC* middle frontal cortex, *NDP* neuronal degeneration phase, *STRI* striatum, *TEC* transentorhinal cortex, *VC* visual cortex. Panel **a**: ln GM tau burden indicates the percentage area occupied (%AO) by AT8-positive pixels in GM after natural log transformation. Least-square means and 95% confidence intervals from the linear mixed-effect model are portrayed

Investigating specific regional patterns of tau burden and neuronal degeneration in each isoform group, we found the highest GM tau burden in the STRI (least-square mean = 1.55, SE = 0.45) in the 3R isoform group; in the STRI (least-square mean = 1.88, SE = 0.50), closely followed by IPL (least-square mean = 1.69, SE = 0.51), MFC (least-square mean = 1.65, SE = 0.50) and ACG (least-square mean = 1.64, SE = 0.52) in the 3R + 4R isoform group; in the TEC (least-square mean = 1.51, SE = 0.33), ATC (least-square mean = 1.48, SE = 0.36) and FG (least-square mean = 1.42, SE = 0.33) in the 4R isoform group (Fig. 5a). This was, to some extent, discordant with the severity of neuronal degeneration (Fig. 5b). The most severe NDP score (i.e. NDP 4) was found in the ATC in all isoform groups (of all ATC assessments: 3R = 85.7%; 3R + 4R = 66.7%; 4R = 29.4%), followed by the TEC and FG in the 3R group (66.7% of all TEC or FG assessments), by the ACG in the 3R + 4R groups (50.0% of all ACG assessments), and by the FG in the 4R group (21.1%). There was some variability in the regional distribution of tau burden and neuronal degeneration in specific *MAPT* variants (Supplementary Fig. 11–12, online resource).

Additionally, we looked specifically at three variants that have been described to have specific frontotemporal atrophy patterns antemortem: R406W and IVS10 + 16 (both primarily temporal, including medial temporal cortices) and P301L (frontotemporal, including lateral temporal, ventromedial prefrontal and anterior cingulate cortices) [78, 85]. Our neuronal degeneration assessment reflected these antemortem atrophy patterns (Supplementary Fig. 13, online resource). In R406W and IVS10 + 16 variants, temporal regions, including medial temporal regions TEC and EC, were most prominently involved, while frontal regions were relatively spared. In P301L, medial temporal regions were less affected than the inferolateral temporal regions FG and ATC, and there was prominent involvement of the medial frontal areas (i.e. FBC, ACG).

### Between-group comparisons of tau burden in different isoform groups

**Fig. 6:**
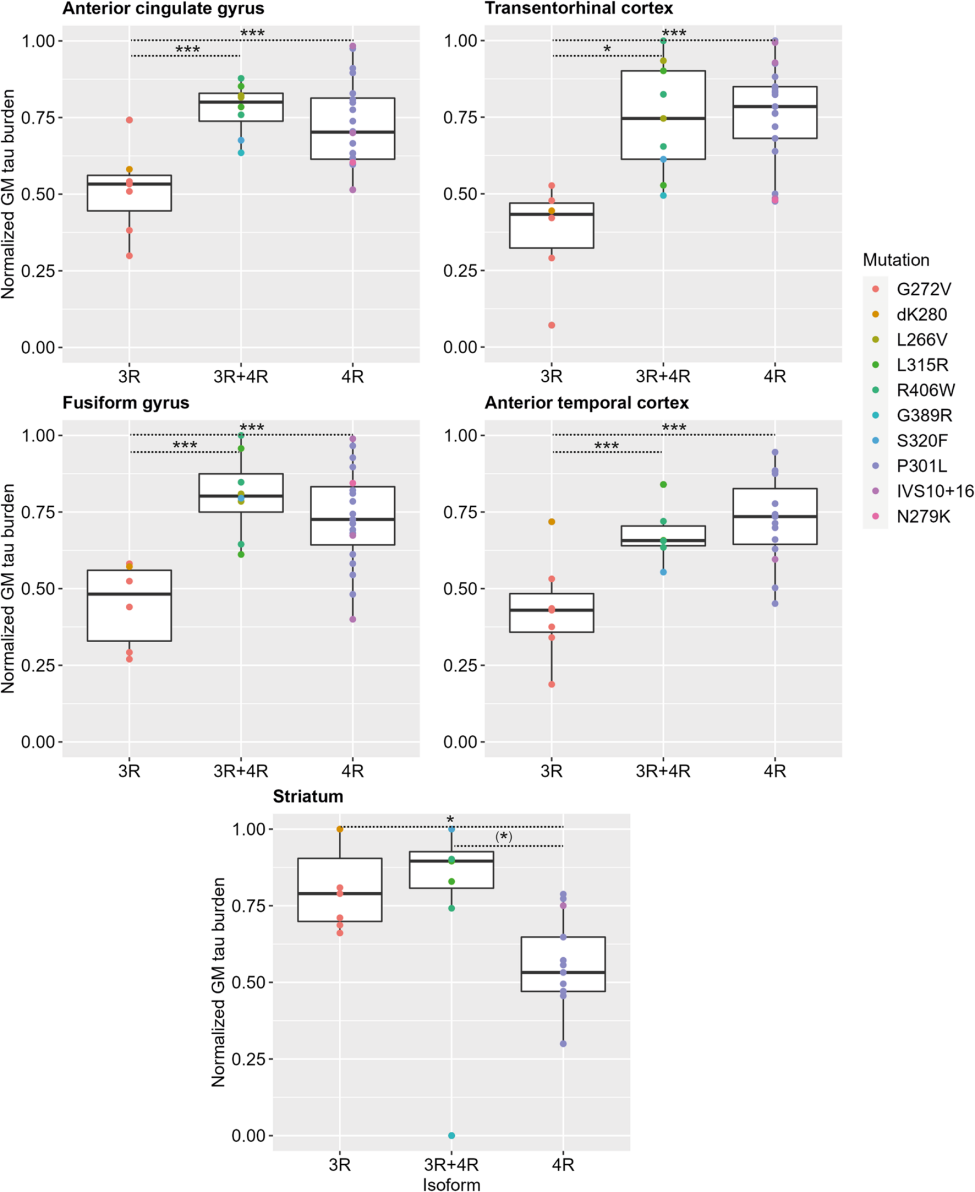
Direct comparisons of grey matter tau burden between isoform groups. Between-group comparisons of normalized GM tau burden showed significant differences between isoform groups in all five most affected regions (*p* < 0.001 for anterior cingulate, anterior temporal, fusiform and transentorhinal cortices; *p* = 0.021 for the striatum). While in the four cortical regions the 3R isoform group had lower GM tau burden than the 3R + 4R and the 4R groups, in the striatum the 3R group had relatively high GM tau burden, similar to the 3R + 4R group and higher than the 4R group (*p* = 0.050). Legend: GM = grey matter. ***p < 0.01. *p ≤ 0.05. (*)*p* < 0.07

We tested for regional group differences in the five most affected regions (i.e. ATC, ACG, FG and TEC with the highest neuronal degeneration and tau burden overall; STRI with the highest tau burden in specific isoforms, i.e. 3R/3R + 4R) by comparing normalized GM tau burden to account for morphological differences in inclusion types between isoform groups. We found significant differences in normalized GM tau burden between isoform groups in all four regions (*p* ≤ 0.001 for ACG, ATC, FG, TEC; *p* = 0.021 for STRI; Fig. 6). Post hoc comparisons showed that the 3R group had significantly less GM tau burden than the 3R + 4R and 4R groups in ACG, ATC, FG and TEC (*p* < 0.013). Conversely, in the STRI, the 3R group showed greater tau burden than 4R (*p* = 0.050) and there was a trend for greater burden in the 3R + 4R group compared to the 4R group (*p* = 0.069).

## Discussion

The current study of the largest reported autopsy cohort of FTLD-MAPT, including ten different *MAPT* variants, enabled to characterize neuronal degeneration and digitally quantified GM tau burden across FTLD-MAPT and in distinct isoform groups. We showed that across the full FTLD-MAPT cohort, tau burden gradually increases through neuronal degeneration phases until it plateaus or declines at (very) severe stages of neuronal degeneration. Notably, the isoform groups showed differences in overall and regional neuropathological severity. Across all regions, the 3R group showed overall lowest tau burden, with the lowest amounts of neuronal and glial tau inclusions, while this group was more frequently associated with severe neuronal degeneration. Conversely, the 4R isoform group showed greater tau burden, of both neuronal and glial tau inclusions, but less severe neuronal degeneration compared to the 3R group. The 3R + 4R isoform group had an intermediate profile with relatively high tau burden (higher than that in 3R), characterized by prominent glial tau inclusions, as well as relatively severe neuronal degeneration (more severe than that in 4R). Isoform groups showed slightly divergent regional patterns of tau burden, which was highest in the striatum in the 3R isoform group, in the striatum and frontoparietal regions in the 3R + 4R group, and in the anterior and medial temporal regions for the 4R group. Yet, most severe neuronal degeneration was consistently found in the anterior temporal cortex within all isoform groups. These findings suggest that the cerebral patterns of tau pathology are somewhat divergent in different tau isoforms, but all have a shared pronounced vulnerability of the anterior temporal cortex to neurodegeneration.

The neuropathological classification of *MAPT* variants has been challenging due to the heterogeneity of neuropathological features in this group [22, 25, 43]. Here, we used a biochemical approach to *MAPT* case classification based on the predominant isoform of tau inclusions [45]. This approach was chosen on the grounds that alterations in 3R:4R isoform ratios are centrally involved in the neurodegenerative process [7, 64, 67], associated with specific *MAPT* variants [22, 25] as well as classes of sporadic tauopathies [21, 43], and are relevant for postmortem diagnostics of tauopathies [34, 43]. Others have chosen to group *MAPT* variants based on morphological classes of sporadic tauopathies (i.e. PiD, CBD, GGT, PSP), arguing that it provides a direct means to compare genetic and sporadic tauopathies [22]. However, here we chose not to classify cases in this manner, since this approach may not be sufficiently comprehensive of all heterogeneous phenotypes of genetic tauopathies, due to the high morphological variability of these variants (Fig. 1). Indeed, while we observed that some individual variants had morphological findings that resembled sporadic tauopathies (i.e. L266V, L315R with sporadic PiD, a subset of P301L with sporadic GGT; Table 2), most individual patient samples and variant groups included additional various glial and neuronal morphologies that did not entirely match findings typical for sporadic tauopathies [13, 43]. Finally, there is limited comparative study of isoform-type groupings of FTLD-MAPT, due in part to the rarity of the disorder, especially for autopsy tissue. Thus, we used our relatively large multicentre cohort to uniquely examine tau burden and neuronal degeneration in FTLD-MAPT using this approach.

The relationship between microscopic neuronal degeneration and tau pathology postmortem can be informative to better understand disease patterns in tauopathies. Most prior autopsy studies of tauopathies examined neuronal degeneration and tau burden as two distinct, parallel measures of postmortem disease [22, 35, 66, 68], or focused mainly on the burden of tau pathology [19, 46, 59]. There has been limited study of how these two measures relate to each other, which may be particularly helpful to understand how atrophy patterns in vivo relate to tissue proteinopathy. Here, we characterized cortical neuronal degeneration with a semi-quantitative five-phase system (i.e. 0–4) based on a set of interrelated parameters, including cortical lamination, neuronal density, vacuolation, as well as NeuN reactivity as an additional supporting criterion (Supplementary Fig. 4, online resource). We further adapted this scale to the assessment of the striatum as a subcortical region, excluding the parameter of cortical lamination (Supplementary Fig. 5, online resource). This approach, which has the advantage of integrating these highly interrelated disease measures into one single score, shows optimal interrater reliability. Additionally, it enabled us to specifically identify samples with extremely severe neuronal degeneration (i.e. NDP 4), which are not easily captured by a three-point ordinal score system such as conventional ratings of neuronal loss (i.e. 0–3). This is particularly useful in FTLD-MAPT that often presents with very severe (focal) atrophy [9].

By modelling the relationship between tau burden and neuronal degeneration in FTLD-MAPT, we found an overall positive association, yet with a slight decrease in tau burden at severe stages of neuronal degeneration (NDP 3–4). Prior studies found that semi-quantitative scores of tau pathology correlate with postmortem neuronal loss in a small group of FTLD-MAPT (*N* = 8, including 3 IVS10 + 16, 2 P301L, 1 V337M, 2 A152T) and in PiD [35, 48], as well as with antemortem volume loss in sporadic tauopathies [66]. Here, we modelled this relationship with increased granularity using digitally measured tau burden, thereby enabling to identify a slight decrease in tau burden at severe stages of neuronal degeneration. We hypothesize that this finding is related to severe tissue and cellular disruption in these end-stage cases, since in FTLD-Tau “ghost” pathology left behind from degenerated neurons is rarely present, unlike in Alzheimer’s disease [37, 61]. These data improve our understanding of the complex relationship between these two microscopic measures, which is important for the development of more accurate (composite) measures of pathological burden and may enable future staging attempts in FTLD-MAPT as well as better comparison of in vivo structural imaging findings with postmortem pathology.

Generalization across all tau isoforms of FTLD-MAPT should, however, be interpreted cautiously, since we also found an important double dissociation between tau burden and neuronal degeneration in different isoform groups. Specifically, the 3R group showed relatively low tau pathology burden, lower than that in the 4R isoform group, while it had overall more severe neuronal degeneration than the 4R group. Additionally, the 3R group also showed the sharpest decline in tau burden at very severe neuronal degeneration phases (Fig. 3c), suggesting that relatively small amounts of 3R tau inclusions may be sufficient to cause very severe neuronal degeneration. Indeed, studies in model systems suggest that oligomeric or other pre-filamentous forms of tau aggregations may be more toxic than the inclusions themselves [29], which could play a role in the 3R group. An alternative interpretation is that tau inclusions may be depleted in these cases due to severe loss of neurons. Interestingly, the 3R + 4R group also showed relatively severe neuronal degeneration, more severe than that in the 4R group. These findings put forward the hypothesis that 3R tau may be more toxic to neurons than 4R tau inclusions alone. Conversely, in the 4R group, relatively high tau burden may be indicative of overall less severe neuronal degeneration, prior to neuronal inclusions’ depletion at severe neuronal degeneration stages. Further, most 4R and 3R + 4R variants showed a relative abundance of glial tau inclusions (Fig. 1, Table 2), which appear less susceptible to depletion at severe neuronal degeneration stages than neuronal inclusions, and may thus in part explain high levels of tau burden in these groups. Our results of more severe neuronal degeneration in 3R tau also align with observations on neuronal loss in sporadic forms of FTLD-Tau. Indeed, sporadic PiD, a 3R-predominant tauopathy, shows severe focal (knife-edge) atrophy in vivo as well as very severe neuronal loss postmortem [9, 56, 79], while other sporadic 4R tauopathies may present relatively less severe atrophy [31, 56]. Thus, there may be substantial differences in how tau isoforms’ inclusions behave and affect the surrounding tissue, but comparisons of *MAPT* to sporadic disease should be explored in detailed future studies. Interestingly, it has been shown in vitro aggregation assays that 4R tau has a greater propensity to aggregate compared to 3R tau [87]. Our findings suggestive of differential neuronal toxicity by tau isoform are limited by the cross-sectional view of human autopsy data on this dynamic process, which should thus be further investigated and coupled with models of tau propagation in in vitro and animal models of tau pathology.

Isoform groups also differed in the relative severity of neuronal and glial tau pathology. While the 3R + 4R group had the highest levels of glial pathology amongst the isoform groups, and the 4R of neuronal pathology, the 3R group had low neuronal and glial pathology, consistent with overall low amounts of tau burden present in these cases (Fig. 3a). These features may in part be associated with specific *MAPT* variants, as observed in our variant-specific subanalysis (Supplementary Fig. 10, online resource) and in line with prior reports describing preferential neuronal and/or glial involvement in specific variants [25]. Indeed, low glial tau pathology in the 3R group may in part be due to the large representation of G272V cases, which have minimal glial pathology [4], while the 3R + 4R group comprises variants that have been associated with widespread astrocytic pathology such as L315R [73], R406W [22, 49] and L266V [32, 72]. Even within specific *MAPT* variants, however, there may be variability in the morphology and relative severity of specific glial or neuronal inclusions in each case, as previously described [22, 25] and observed in this study (Table 2).

The regional patterns of tau burden diverged to some extent between isoform groups of FTLD-MAPT. Across the full cohort, FTLD-MAPT was characterized by elevated GM tau burden and neuronal degeneration in anterior, inferior and medial temporal and anterior cingulate regions. This is consistent with prior postmortem studies (see prior review [25]), as well as antemortem studies pointing to a most prominent involvement of the temporal lobe as well as medial frontal regions in FTLD-MAPT [56, 77, 78, 81], even at presymptomatic or early stages [5, 6, 8, 10, 14, 82]. Here, in addition, we found distinct regions of greatest GM tau burden in each isoform group, which may suggest somewhat divergent spread of tau pathology. Anterior and medial temporal regions were associated with greatest tau burden in 4R variants, including the relatively large groups of P301L and IVS10 + 16. On the other hand, the striatum had the highest tau burden in the 3R and 3R + 4R groups, next to frontoparietal regions in the 3R + 4R group. Remarkably, high tau burden in the striatum emerged as a distinctive feature in 3R and 3R + 4R isoforms, as opposed to the 4R isoform (Fig. 6). Interestingly, in the 3R group striatal tau burden was strikingly higher than that in other cortical regions, while neuronal degeneration in this region was not as severe as in other cortical regions (ATC, FG, ACG, TEC). This may suggest a propensity for the accumulation of tau inclusions in this subcortical region, along with a somewhat lower vulnerability to neuronal degeneration. It may be the case that the striatum becomes affected relatively later in the disease course, and therefore does not show (yet) the combination of very low tau burden and extreme tissue degeneration observed in other cortical regions. However, it is difficult to make definitive conclusions on the time sequence and progression of tauopathy and resultant neurodegeneration from cross-sectional autopsy data alone. While we adjusted for neuronal degeneration in these analyses to partly account for this in the interpretation of regional tau burden, we lack established histopathological staging systems to help contextualize our findings in the temporal sequence of spreading pathology. Future work in larger samples with antemortem tau imaging may be able to develop staging patterns of progressive tauopathy across isoform groups.

Despite differing distribution of tau burden in isoform groups, neuronal degeneration was most severe in the anterior temporal cortex in each isoform group. This partial dissociation between tau burden and neuronal degeneration suggests that tau pathology may distribute or spread through different regional nodes within frontotemporal paralimbic networks in different isoform groups, yet the anterior temporal cortex may have an increased vulnerability to neuronal degeneration despite varying severity of tau inclusions. A recent imaging study across presymptomatic and symptomatic carriers found distinct atrophy patterns associated with specific *MAPT* variants, i.e. a temporal pattern including mesiotemporal structures associated with IVS10 + 16 and R406W variants, and a frontotemporal pattern including (lateral) temporal and ventromedial regions associated with the P301L variant [84, 85]. These patterns in part reflect our findings of neuronal degeneration in the corresponding isoform groups, with the involvement of the antero-inferior temporal cortex across all isoform groups and of the medial temporal cortex in the 3R + 4R group (Fig. 5b), and are further supported by our findings of neuronal degeneration severity in subanalyses looking at these specific *MAPT* variants (Supplementary Fig. 13, online resource). These subtle differences between specific variants, captured through in vivo whole-brain analyses [84, 85], should be further validated in postmortem studies focused on larger groups of specific *MAPT* variants as well as in studies investigating tissue-imaging correlations.

The distribution of pathology in FTLD-MAPT isoform groups differs, to some extent, from that observed in FTLD-spectrum sporadic tauopathies with the same predominant isoform, which can give rise to similar clinical presentations. In our study, the 3R FTLD-MAPT group showed the greatest tau burden in the striatum, while the 3R + 4R group showed relatively high tau burden in frontal, cingulate and inferior parietal areas, next to the striatum. Comparatively, in a sporadic FTLD-associated 3R tauopathy such as PiD, the most severe postmortem tau burden was observed in the medial temporal regions, i.e. the amygdala, the (para)hippocampus and the inferior temporal gyrus, along with the paralimbic anterior cingulate cortex, while the striatum is less affected [35]. On the other hand, patterns of neuronal degeneration appear relatively similar between sporadic and genetic 3R tauopathies. In both 3R and 3R + 4R FTLD-MAPT, we found moderate to severe neuronal degeneration of the anterior temporal, anterior cingulate and transentorhinal cortices, which may thus be regions highly vulnerable to neurodegeneration for 3R tau. Similarly, in sporadic PiD, most severe neuronal degeneration postmortem is found in the inferomedial temporal regions [83]. While the anterior temporal lobe has been understudied in autopsy studies, MRI studies of autopsy-confirmed PiD have shown involvement of this region [56, 80]. On the other hand, in 4R FTLD-MAPT we found the greatest tau burden and neuronal degeneration in the anteromedial temporal cortex. Differently, in FTLD-associated 4R sporadic tauopathies, such as PSP and CBD, tau pathology tends to affect the frontoparietal areas cortically, with relatively less severe involvement of the temporal lobe [26, 46]. These discrepancies suggest different mechanisms underlying disease spread and progression between sporadic and genetic tauopathies despite similar clinical presentations. However, these comparisons should be interpreted with caution, due to the differences in pathological phenotypes, and possibly in tau filament structure, between genetic and sporadic subtypes. While filament structures detected through cryo-electron microscopy studies have been described for FTLD-associated sporadic tauopathies [16, 62, 86], as well as for Alzheimer’s disease and chronic traumatic encephalopathy [17, 18, 20, 60], the tau structure of most genetic tauopathies is still unknown. Future studies investigating the structure of tau fibrils implicated in genetic tauopathies may reveal isoform- or variant-associated tau strains, which will facilitate the identification of similarities and differences between genetic and sporadic tauopathies at the structural and pathophysiological level.

Some limitations should be considered when interpreting the findings of this study. While we examined the largest cohort of FTLD-MAPT described thus far, the number of variants included is only a subset of all reported variants (> 60) and, due to the small number of cases with specific variants, we were unable to perform subanalyses comparing variant groups. Because of the postmortem nature of this study, including cases of autopsy in the past three decades, some clinical data and brain tissue were missing, which we partly accounted for statistically using mixed modelling in pathology analyses. Co-pathology of postmortem tissue in this relatively young-onset cohort was limited, and likely did not influence the results (Table 1). We included a selection of regions, which were sampled in a standardized manner across the two centres. As we investigated the striatum as a single subcortical region, we cannot exclude that other subcortical regions may provide additional information in the study of FTLD-MAPT pathology. Further, as tissue was sampled unilaterally in most cases, we had insufficient bilateral data to perform laterality analyses and we may have missed hemispheric differences that are possible in FTLD-MAPT. However, covarying for hemisphere in our analyses did not reveal a significant effect on the results, and gross macroscopy findings at autopsy indicated asymmetry in only a small percentage of cases (2/38, 5.3%). While we used rigorous and validated methods for digital quantification of tau burden, neuronal degeneration was assessed semi-quantitatively. NDP scores showed optimal reliability in validation analyses, but they are relatively less suitable for statistical modelling than quantitative scores; future studies should attempt to identify reliable measures to quantify neuronal degeneration postmortem. Additionally, other measures such as microgliosis, astrogliosis and axonal demyelination may be helpful to gain a more complete understanding of complex disease dynamics. Finally, further study of the biochemical and structural properties of tau aggregates may provide valuable insights into the heterogeneity of FTLD-MAPT, as other differences next to tau isoforms are important for disease mechanisms, such as tau conformation, post-translational modifications, truncation, and aggregation [67]. For instance, recent findings in AD link distinct tau conformers to a more rapid disease progression in AD [40] and post-translational modification to different disease stages of AD [76], and specific tau filament folds observed with cryo-electron microscopy have been associated with specific classes of sporadic tauopathies [16, 62, 86], laying the foundation for a structure-based classification of 3R and 4R tauopathies. Follow-up studies looking at these biochemical and structural features in FTLD-MAPT are therefore needed. Increased understanding of 3R and 4R tau isoform groupings may have implications for the antemortem diagnosis of tauopathies. For fluid biomarkers, assays to detect 3R and 4R tau aggregates through RT-QuiC technology are currently being developed and validated in different tau isoforms [15, 52, 58, 69]. As for tau PET, current tracers find most affinity for 3R + 4R tau, such as R406W and V337M mutations in FTLD-MAPT [39, 47, 63, 71, 82]. Development of novel tau tracers to identify the full spectrum of 3R, 4R and 3R + 4R tau are needed in FTLD-MAPT and sporadic FTLD-Tau.

In this postmortem study of FTLD-MAPT, we show that postmortem disease heterogeneity can in part be explained by the predominant tau isoform, which seems to influence the severity of tau burden and neuronal degeneration. Particularly, 3R variants are associated with lower tau burden, but more severe neuronal degeneration, whereas 4R variants show greater (neuronal and glial) tau burden but less severe neuronal degeneration, suggesting that 3R tau may be more toxic to neurons than 4R tau. Further, while isoform groups in part diverge in their distribution of tau burden, with 3R and 3R + 4R variants more likely to affect the striatum, along with frontoparietal regions in 3R + 4R variants, and 4R variants anteromedial temporal regions, we find in each isoform group a pronounced vulnerability of the anterior temporal cortex to neuronal degeneration.

## Supplementary Information

Below is the link to the electronic supplementary material.

Supplementary file1 (DOCX 5057 kb)

## Data Availability

Data from this study will be made available by the corresponding authors upon reasonable request.
