# Correction to: Isoform-specific patterns of tau burden and neuronal degeneration in *MAPT*-associated frontotemporal lobar degeneration

**DOI:** 10.1007/s00401-023-02556-2

**Published:** 2023-03-17

**Authors:** Lucia A. A. Giannini, Daniel T. Ohm, Annemieke J. M. Rozemuller, Laynie Dratch, EunRan Suh, Vivianna M. van Deerlin, John Q. Trojanowski, Edward B. Lee, John C. van Swieten, Murray Grossman, Harro Seelaar, David J. Irwin, Netherlands Brain Bank

**Affiliations:** 1 Alzheimer Center, Department of Neurology, Erasmus University Medical Center, Doctor Molewaterplein 40, 3015 GD Rotterdam, The Netherlands; 2 Digital Neuropathology Laboratory, Department of Neurology, Perelman School of Medicine, University of Pennsylvania, Philadelphia, PA 19104 USA; 3 Frontotemporal Degeneration Center (FTDC), University of Pennsylvania Perelman School of Medicine, Hospital of the University of Pennsylvania, 3600 Spruce Street, Philadelphia, PA 19104 USA; 4 Department of Pathology, Amsterdam Neuroscience, Amsterdam University Medical Center, Location VUmc, Amsterdam, The Netherlands; 5 Center for Neurodegenerative Disease Research, Department of Pathology and Laboratory Medicine, Perelman School of Medicine, University of Pennsylvania, Philadelphia, PA 19104 USA; 6 Translational Neuropathology Research Laboratory, Department of Pathology and Laboratory Medicine, Perelman School of Medicine, University of Pennsylvania, Philadelphia, PA 19104 USA

**Correction to: Acta Neuropathologica (2022) 144:1065–1084** 10.1007/s00401-022-02487-4

The article Isoform‑specific patterns of tau burden and neuronal degeneration in MAPT‑associated frontotemporal lobar degeneration, written by Lucia A. A. Giannini, Daniel T. Ohm, Annemieke J. M. Rozemuller, Laynie Dratch, EunRan Suh, Vivianna M. van Deerlin, John Q. Trojanowski, Edward B. Lee, John C. van Swieten, Murray Grossman, Harro Seelaar, David J. Irwin, Netherlands Brain Bank, was originally published Online First without Open Access. After publication in volume 144, issue 6, page 1065–1084 the author decided to opt for Open Choice and to make the article an Open Access publication. Therefore, the copyright of the article has been changed to © The Author(s) 2023 and the article is forthwith distributed under the terms of the “Creative Commons Attribution 4.0 International License, which permits use, sharing, adaptation, distribution and reproduction in any medium or format, as long as you give appropriate credit to the original author(s) and the source, provide a link to the Creative Commons licence, and indicate if changes were made. The images or other third party material in this article are included in the article’s Creative Commons licence, unless indicated otherwise in a credit line to the material. If material is not included in the article’s Creative Commons licence and your intended use is not permitted by statutory regulation or exceeds the permitted use, you will need to obtain permission directly from the copyright holder. To view a copy of this licence, visit http://creativecommons.org/licenses/by/4.0/”.

The original article has been corrected.

