# Supplementary material for: Isoform-specific patterns of tau burden and neuronal degeneration in MAPT-associated frontotemporal lobar degeneration
Source: Acta Neuropathol. 2022 Sep 6;144(6):1065–84. doi: 10.1007/s00401-022-02487-4 (PMC9995405; doi:10.1007/s00401-022-02487-4)
Supplement: Supplementary file 1 — Supplementary file1 (DOCX 5057 kb) [file 401_2022_2487_MOESM1_ESM.docx]

**Online supplement for:**

**Isoform-specific patterns of tau burden and neuronal degeneration in *MAPT*-associated frontotemporal lobar degeneration**

Lucia AA Giannini, MD^1^, Daniel T Ohm, PhD^2,3^, Annemieke JM Rozemuller, MD, PhD^4^, Laynie Dratch, ScM, CGC^3^, EunRan Suh, PhD^5^, Vivianna M van Deerlin, MD, PhD^5^, John Q Trojanowski, MD, PhD^5#^, Edward B Lee, MD, PhD^5,6^, Netherlands Brain Bank, John C van Swieten, MD, PhD^1^, Murray Grossman, MD^3^, Harro Seelaar, MD, PhD^1^*, David J Irwin, MD^2,3^*

^1^Department of Neurology and Alzheimer Center Erasmus MC, Erasmus University Medical Center, Rotterdam, the Netherlands

^2^Digital Neuropathology Laboratory, Department of Neurology, Perelman School of Medicine, University of Pennsylvania, Philadelphia, PA 19104, USA

^3^Penn Frontotemporal Degeneration Center, Department of Neurology, Perelman School of Medicine, University of Pennsylvania, Philadelphia, PA 19104, USA

^4^Department of Pathology, Amsterdam Neuroscience, Amsterdam University Medical Center, location VUmc, Amsterdam, The Netherlands

^5^Center for Neurodegenerative Disease Research, Department of Pathology and Laboratory Medicine, Perelman School of Medicine, University of Pennsylvania, Philadelphia, PA 19104, USA

^6^Translational Neuropathology Research Laboratory, Department of Pathology and Laboratory Medicine, Perelman School of Medicine, University of Pennsylvania, Philadelphia, PA 19104, USA

*These authors contributed equally

^#^posthumous

Running title: Tau and neuronal degeneration in FTLD-MAPT

Please send correspondence to:

*Harro Seelaar, MD, PhD

Alzheimer Center, Department of Neurology

Erasmus University Medical Center

Doctor Molewaterplein 40

3015 GD Rotterdam

h.seelaar@erasmusmc.nl

*David J. Irwin, MD

Frontotemporal Degeneration Center (FTDC)

University of Pennsylvania Perelman School of Medicine

Hospital of the University of Pennsylvania

3600 Spruce Street, Philadelphia, PA 19104

(215)-662-7682

dirwin@pennmedicine.upenn.edu

Supplementary material:

13 Supplementary Figures, 4 Supplementary Tables, 1 Supplementary Methods

## FIGURES

**Supplementary Fig. 1 Digital pathology sampling of the cortex and striatum**


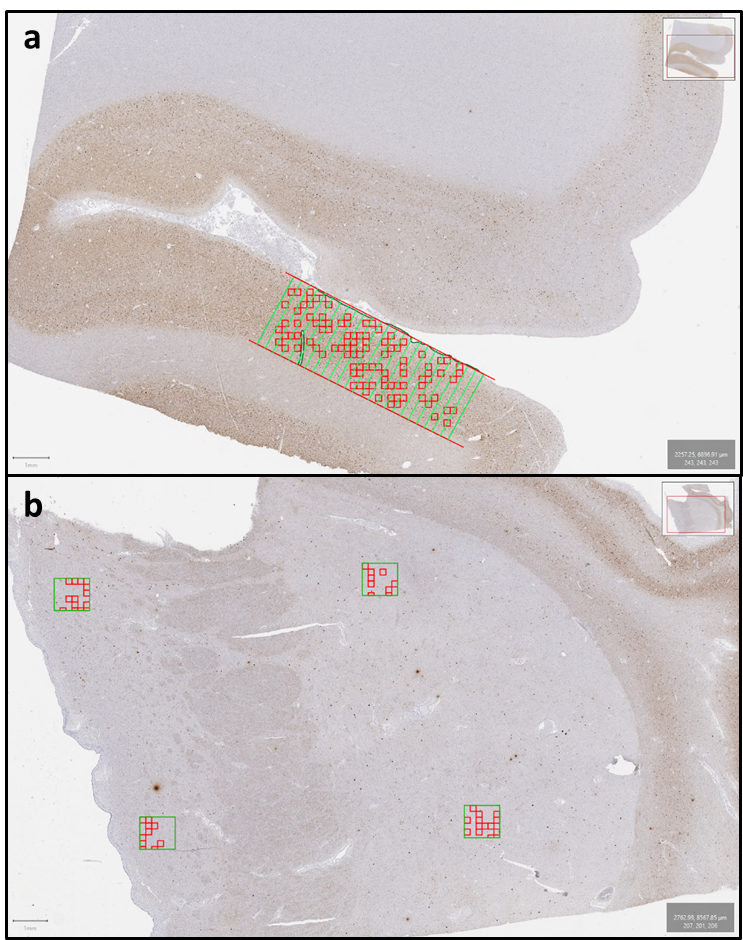


The digital sampling methods for the cortex (a) and the striatum (b) are illustrated. In the cortex, our region of interest (ROI) sampled the longest stretch of parallel-oriented cortex in a tissue section. In case of variability in disease severity within a section, we chose to sample the area with greatest disease severity (i.e. greatest neuronal degeneration; in case this was equal throughout the section, we sampled the area with the greatest tau burden). In the striatum, we placed four square ROIs of 1 mm^2^ evenly distributed throughout the striatal tissue, while being blinded to tau burden. Within each ROI, 30% of the area was randomly selected with 175x175 µm tile annotations (i.e. small red squares). Percentage area occupied by AT8-positive pixels was calculated within the tiles and averaged across all tiles to obtain cortical and striatal measurements of tau burden.

**Supplementary Fig. 2 Bland-Altman plots of %AO measurements from slides stained in duplicates from three staining batches**

**
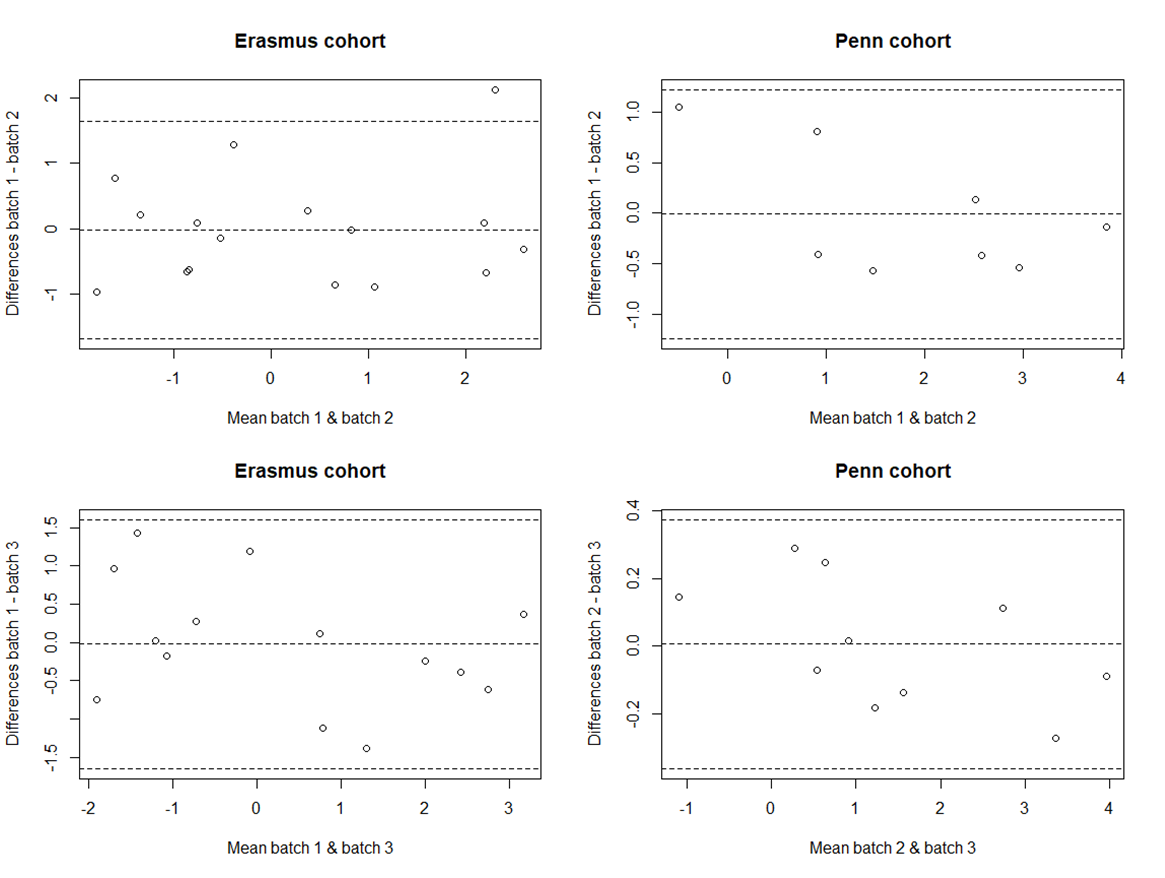
**

Bland-Altman plots show minimal bias approaching zero when comparing %AO measurements from slides stained in duplicate from distinct staining batches in each cohort. Based on availability of slides stained in duplicate, we used Batch 1 as reference batch in the Erasmus cohort, and Batch 2 in the Penn cohort. Bland-Altman comparisons were performed after empirical generation of RGB staining algorithms in each batch and optimization of optical density (OD) values to minimize differences between batches. Bland-Altman statistical testing found no significant bias between the batches (p > 0.9 for all).

**Supplementary Fig. 3 Validation of digital %AO scores using gold-standard ordinal scores**

**
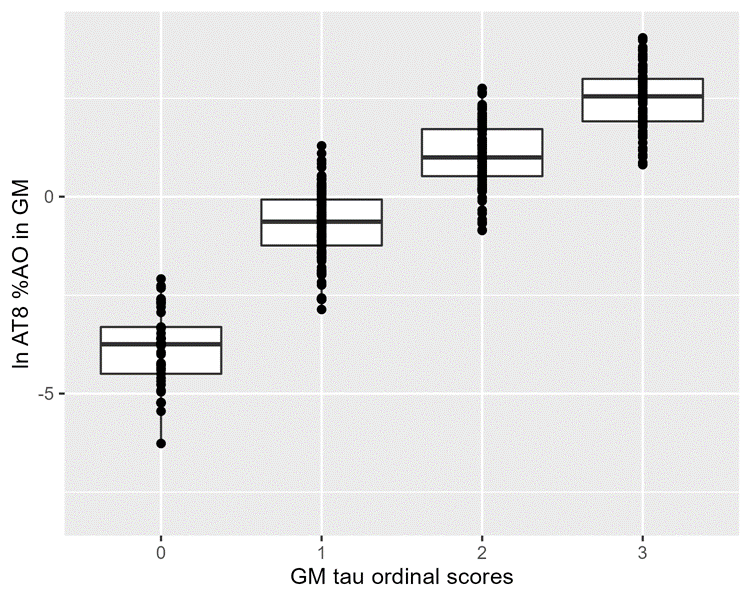
**

Digitally obtained %AO scores, quantifying AT8-positive pixels, were validated by comparison to gold-standard ordinal ratings of GM tau burden (after applying natural logarithmic transformation). Analysis of variance (ANOVA) found significant differences in %AO scores between ordinal score groups (df = 3,326, F = 582.4, p < 0.001), and pairwise t-tests with Bonferroni correction showed significant differences between all ordinal score groups (p < 0.001 for all pairwise comparisons). Legend: %AO = percentage of area occupied; GM = grey matter.

**Supplementary Fig. 4 Cortical neuronal degeneration phases (NDP 0-4)**


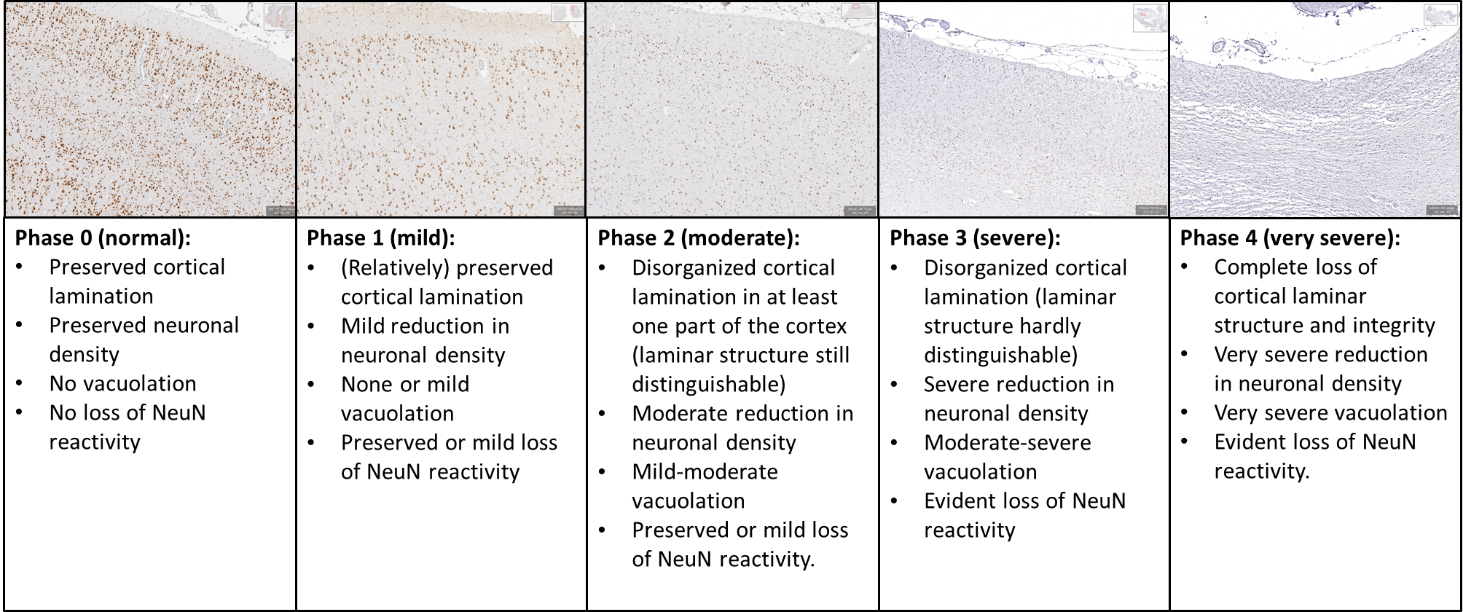
Figure displays our neuronal degeneration phase (NDP) grading scheme based on four parameters: cortical lamination, neuronal density, vacuolation and nuclear NeuN reactivity. Briefly, NDP 0 corresponded to a normal cortex. NDP 1 was defined as mild neuronal degeneration, i.e. a mild reduction in neuronal density in the context of preserved cortical lamination, none or mild vacuolation, preserved or mild loss of nuclear NeuN reactivity. NDP 2 was defined as moderate neuronal degeneration, i.e. moderate disruption of at least one part of the cortex showing disorganized lamination (laminar structure still distinguishable), moderate reduction in neuronal density, mild-moderate vacuolation, and preserved or mild loss of nuclear NeuN reactivity. NDP 3 was defined as severe neuronal degeneration, i.e. severe disruption of the entire cortex showing disorganized lamination (laminar structure hardly distinguishable), severe reduction in neuronal density, moderate-severe vacuolation and evident loss of nuclear NeuN reactivity. NDP 4 was defined as very severe neuronal degeneration, i.e. complete loss of laminar structure and integrity, extreme neuronal depletion and vacuolation, evident loss of nuclear NeuN reactivity.

**Supplementary Fig. 5 Striatal neuronal degeneration phases (NDP 0-4)**


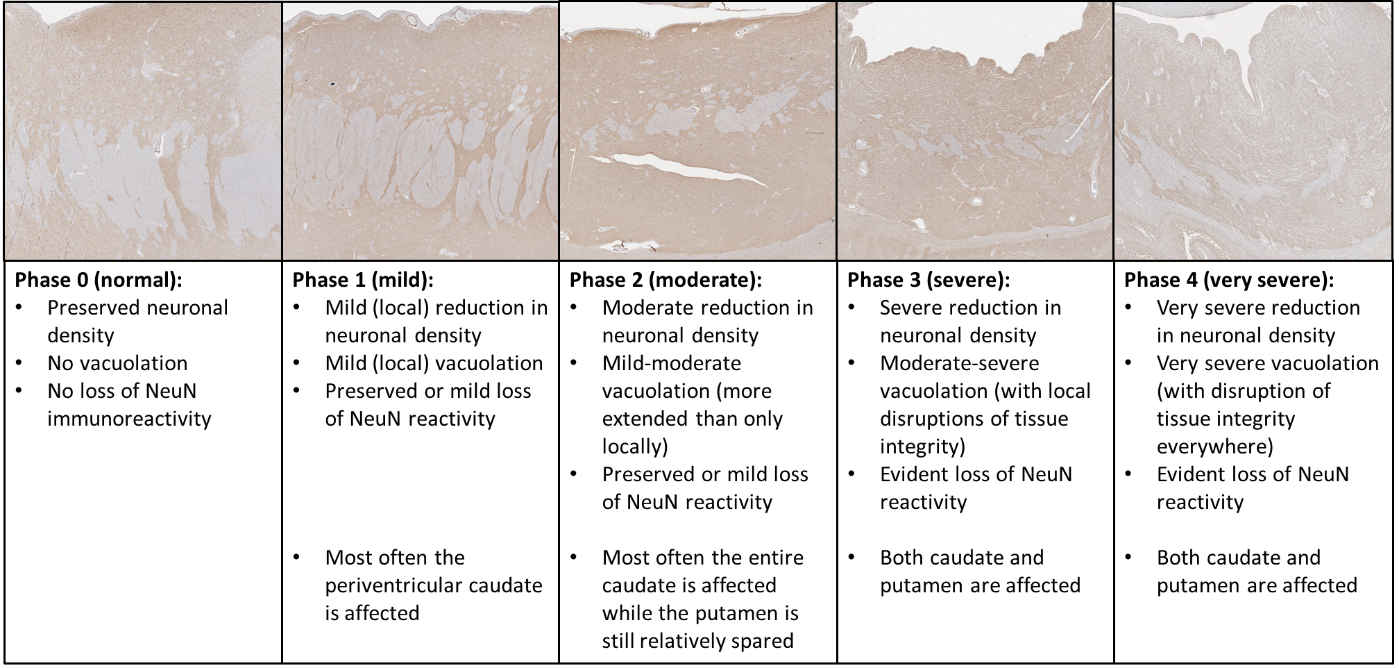
Figure displays the neuronal degeneration phase (NDP) grading scheme adapted to the assessment of the striatum. In this scheme, we did not take into account cortical lamination, while we focused on parameters of neuronal density, vacuolation and nuclear NeuN reactivity. Additionally, our assessment was supported by some anatomical considerations of neuronal degeneration distribution patterns described in the bottom row.

**Supplementary Fig. 6 Validation of neuronal degeneration phases**

**
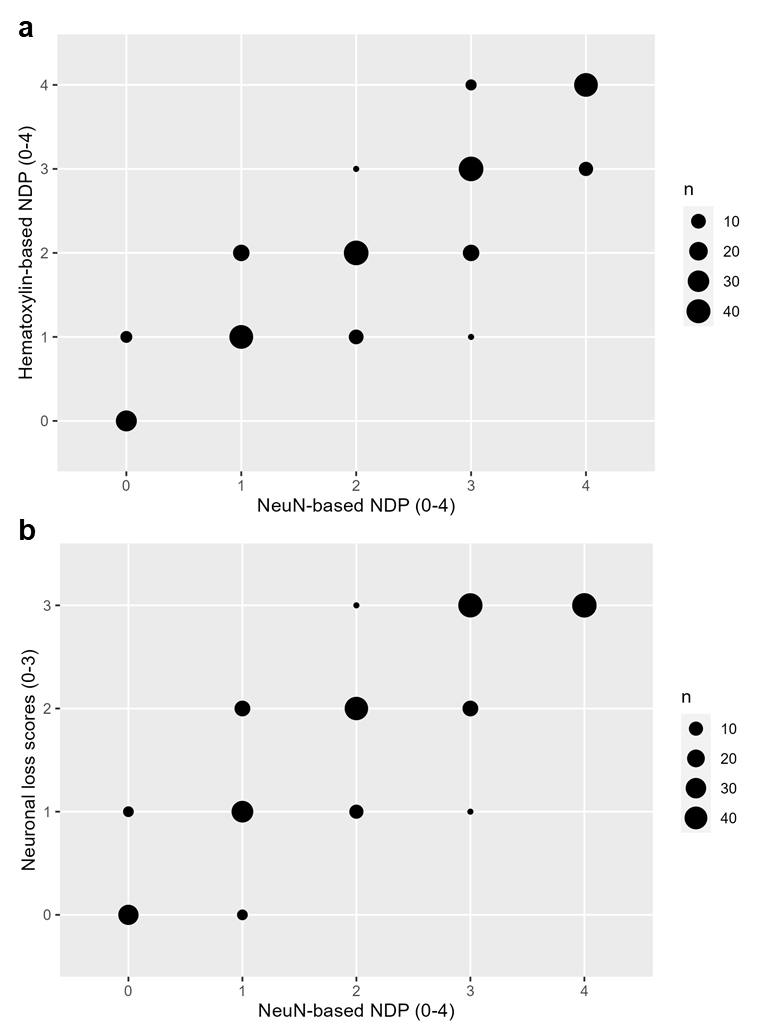
**

(a) NDP scores based on NeuN-stained tissue showed optimal agreement with NDP scores based on hematoxylin-stained tissue (Kappa = 0.84, p < 0.001). Thus, hematoxylin-based NDP scores could be reliably used to supplement missing data points due to missing NeuN-stained tissue. (B) NDP scores were validated by comparison to conventional neuronal loss scores (0-3). Statistical analysis found a strong positive correlation between the two measures (rho = 0.91, p < 0.001). Legend: NDP = neuronal degeneration phase.

**Supplementary Fig. 7 Relation between striatal/cortical neuronal degeneration and tau burden in each isoform group**


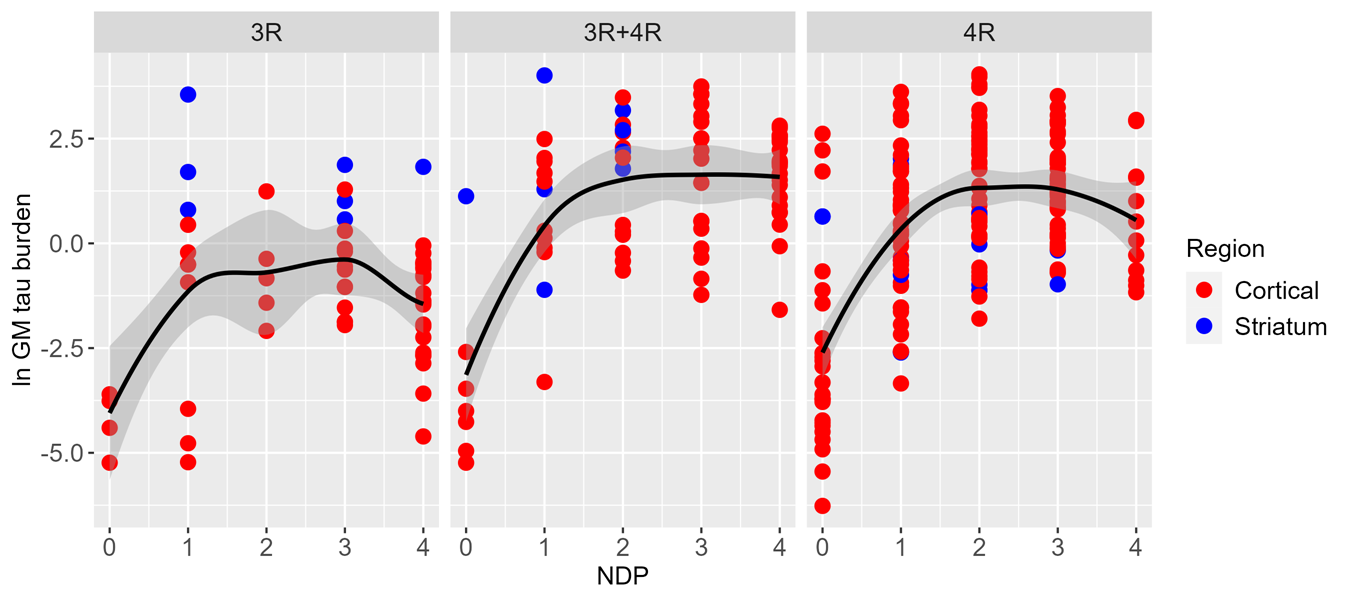


Plot portrays the relationship between neuronal degeneration (i.e. NDP) and GM tau burden in each isoform group, highlighting cortical vs. striatal samples. Legend: GM = grey matter; NDP = neuronal degeneration phase. On the y-axis, ln GM tau burden indicates the percentage area occupied (%AO) by AT8-positive pixels in GM after natural log transformation.

**Supplementary Fig. 8 Tau burden and neuronal degeneration in specific *MAPT* variants**


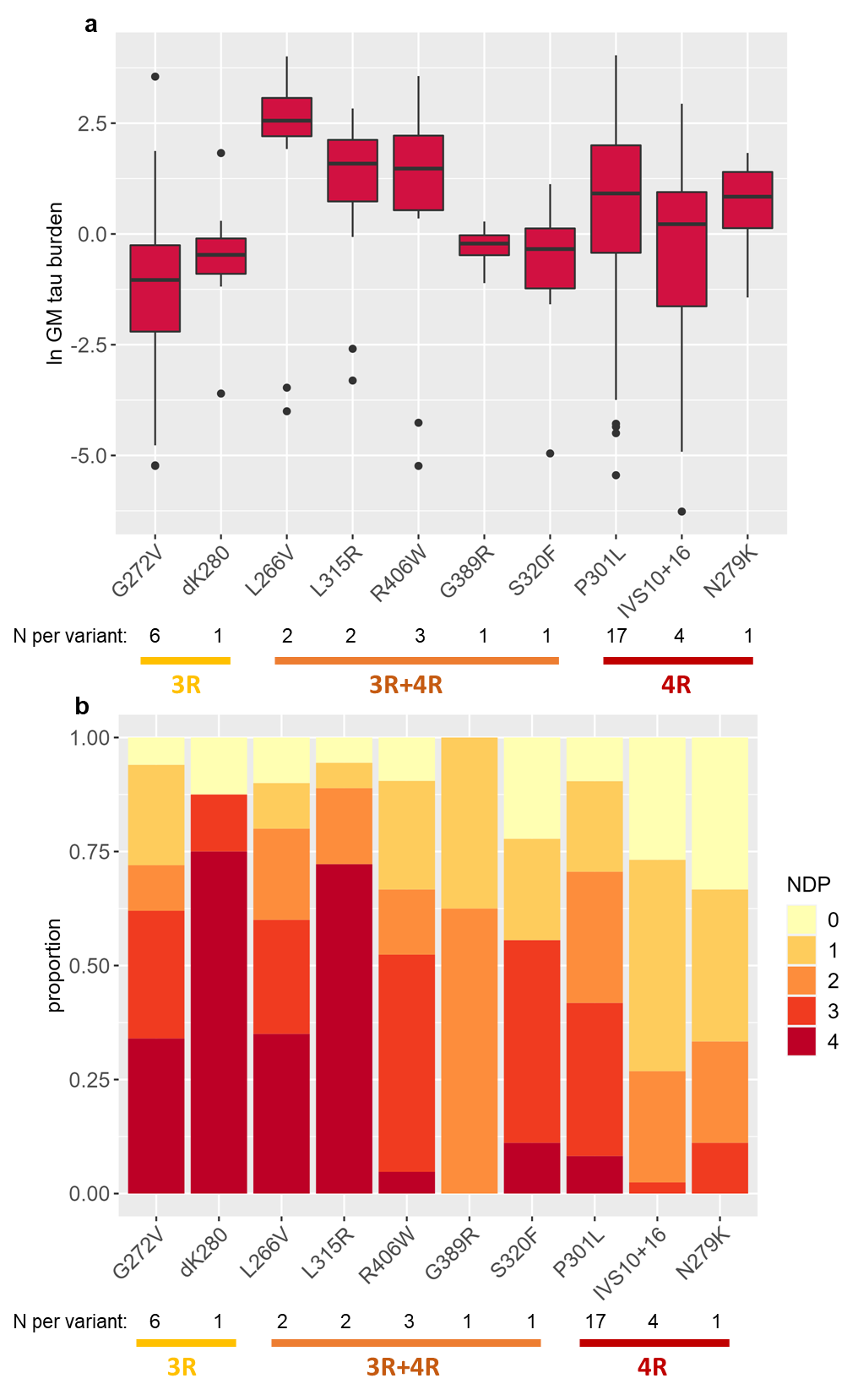


Plots portray the severity of overall tau burden (a) and neuronal degeneration (b) across all sampled regions in each *MAPT* variant. We highlight variants belonging to 3R (yellow), 3R+4R (orange) and 4R (red) isoform groups, and the number of cases (N) for each variant. Legend: GM = grey matter; NDP = neuronal degeneration phase. On the y-axis in panel a, ln GM tau burden indicates the percentage area occupied (%AO) by AT8-positive pixels in GM after natural log transformation.

**Supplementary Fig. 9 Relation between neuronal degeneration and tau burden in each *MAPT* variant**

**
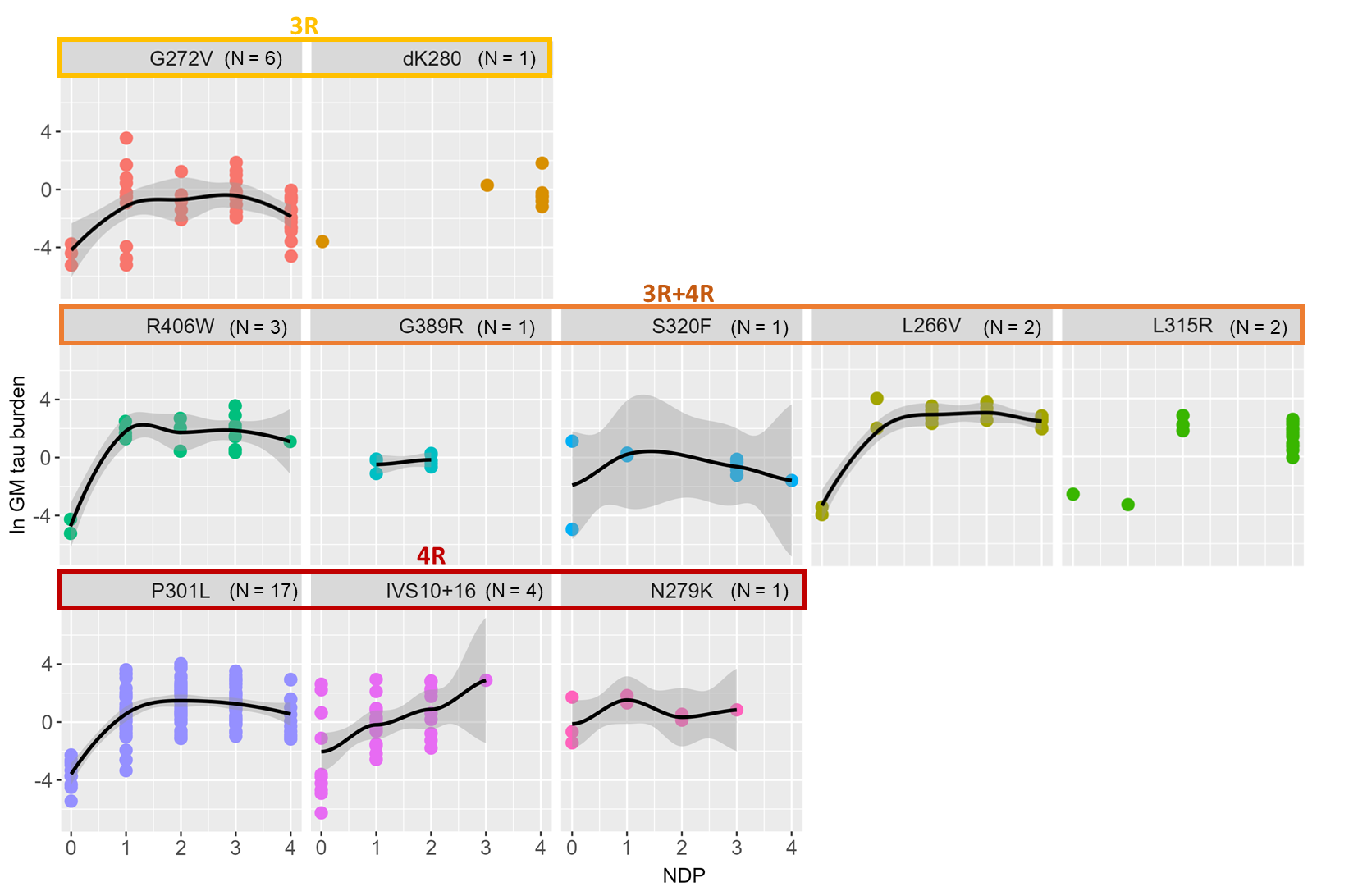
**Plot portrays the relationship between neuronal degeneration (i.e. NDP) and GM tau burden in each *MAPT* variant. We highlight variants belonging to 3R (yellow), 3R+4R (orange) and 4R (red) isoform groups, and the number of cases (N) for each variant. Legend: GM = grey matter; NDP = neuronal degeneration phase. On the y-axis, ln GM tau burden indicates the percentage area occupied (%AO) by AT8-positive pixels in GM after natural log transformation.

**Supplementary Fig. 10 Relative severity of neuronal and glial tau pathology in each *MAPT* variant**

**
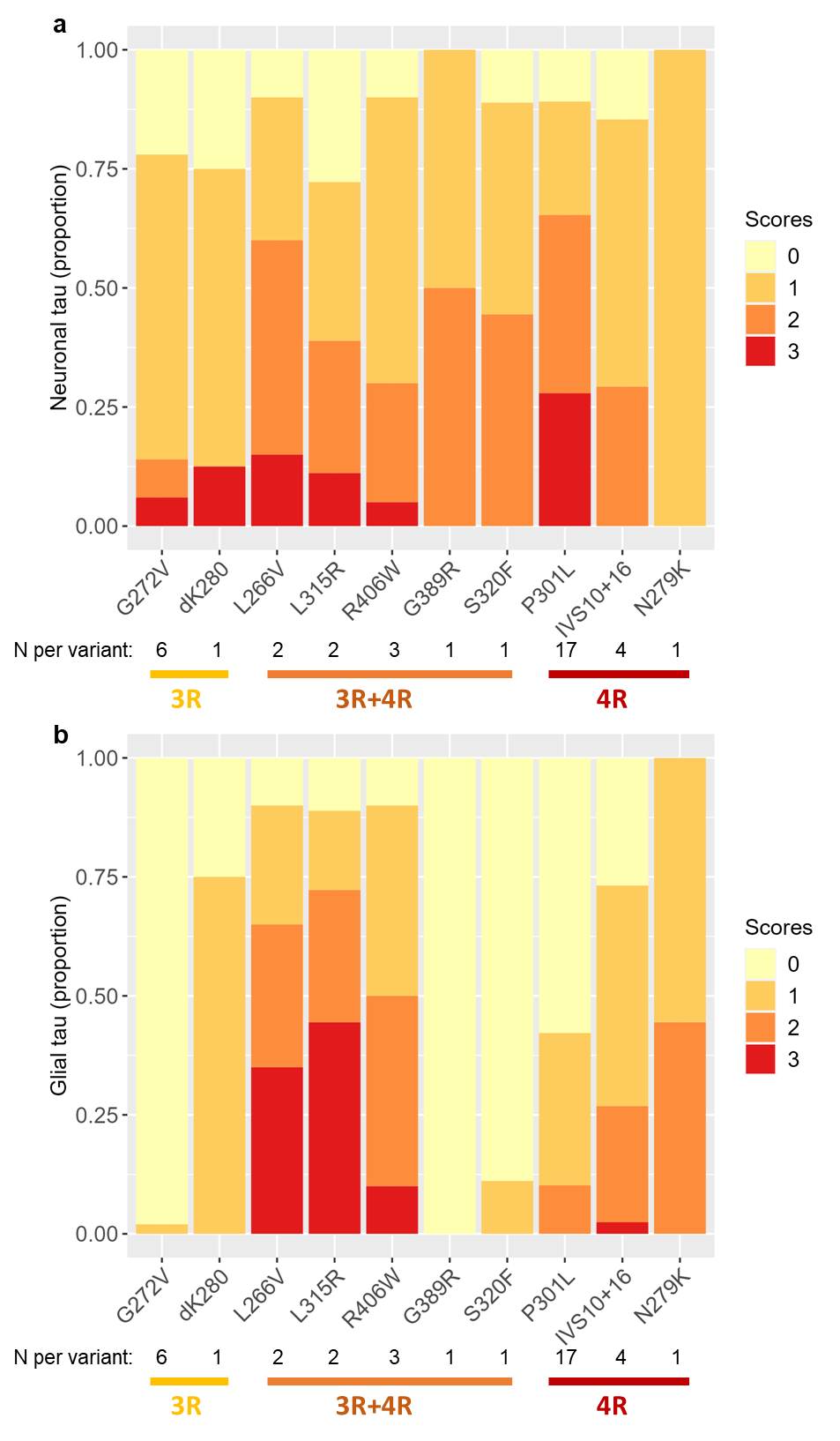
**

Plots portray the relative burden of neuronal (a) and glial (b) tau pathology (i.e. ordinal scores 0-3) across all sampled regions in each *MAPT* variant. We highlight variants belonging to 3R (yellow), 3R+4R (orange) and 4R (red) isoform groups, and the number of cases (N) for each variant.

**Supplementary Fig. 11 Regional distribution of tau burden in each *MAPT* variant**


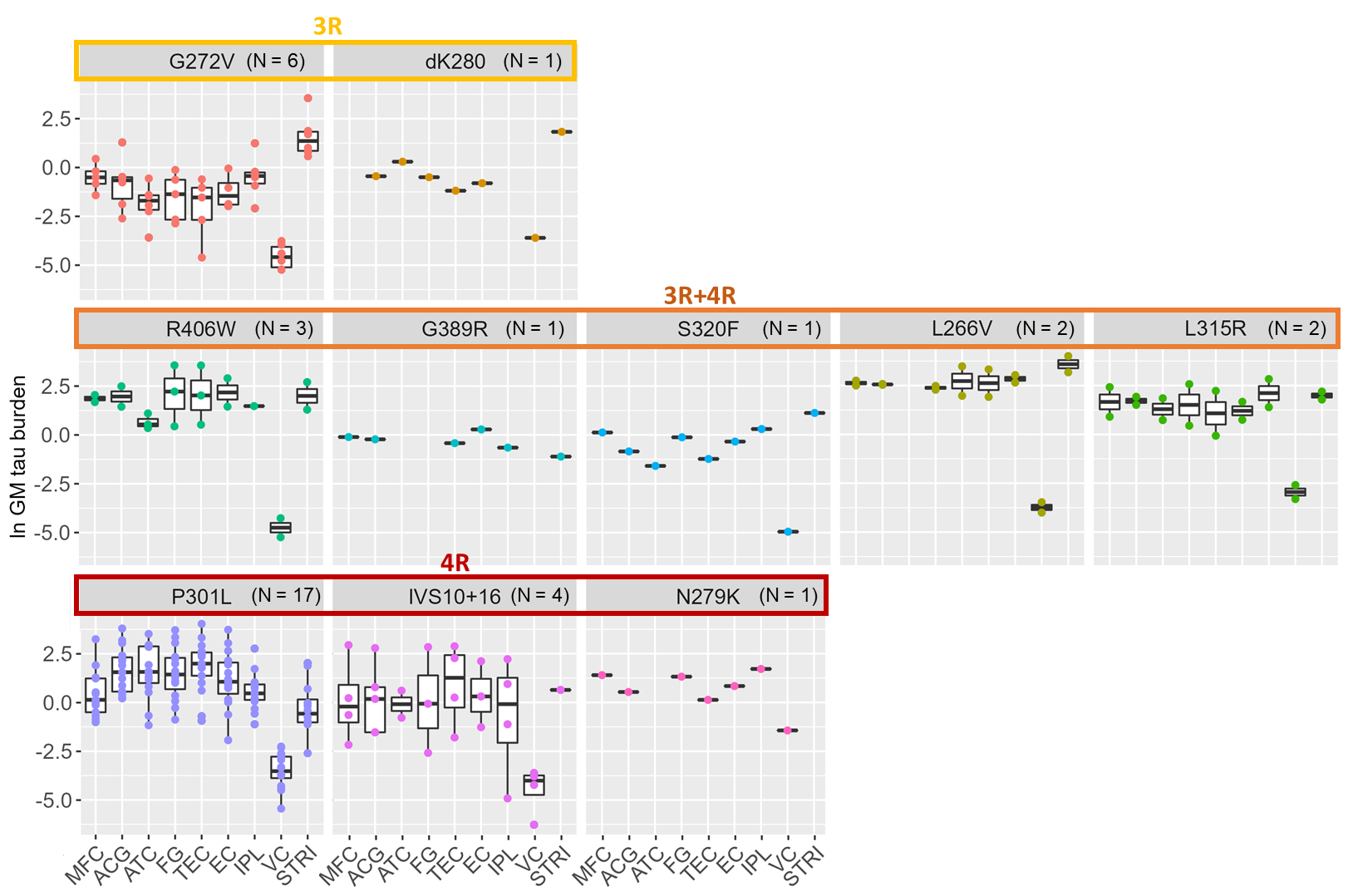


Plots portray the regional distribution of GM tau burden in 9 regions with most available data in each *MAPT* variant. We highlight variants belonging to 3R (yellow), 3R+4R (orange) and 4R (red) isoform groups, and the number of cases (N) for each variant. Legend: ACG = anterior cingulate gyrus; ATC = anterior temporal cortex; GM = grey matter; EC = entorhinal cortex; FG = fusiform gyrus; IPL = inferior parietal lobule; MFC = middle frontal cortex; STRI = striatum; TEC = transentorhinal cortex; VC = visual cortex. On the y-axis, ln GM tau burden indicates the percentage area occupied (%AO) by AT8-positive pixels in GM after natural log transformation.

**Supplementary Fig. 12 Regional distribution of neuronal degeneration in each *MAPT* variant**


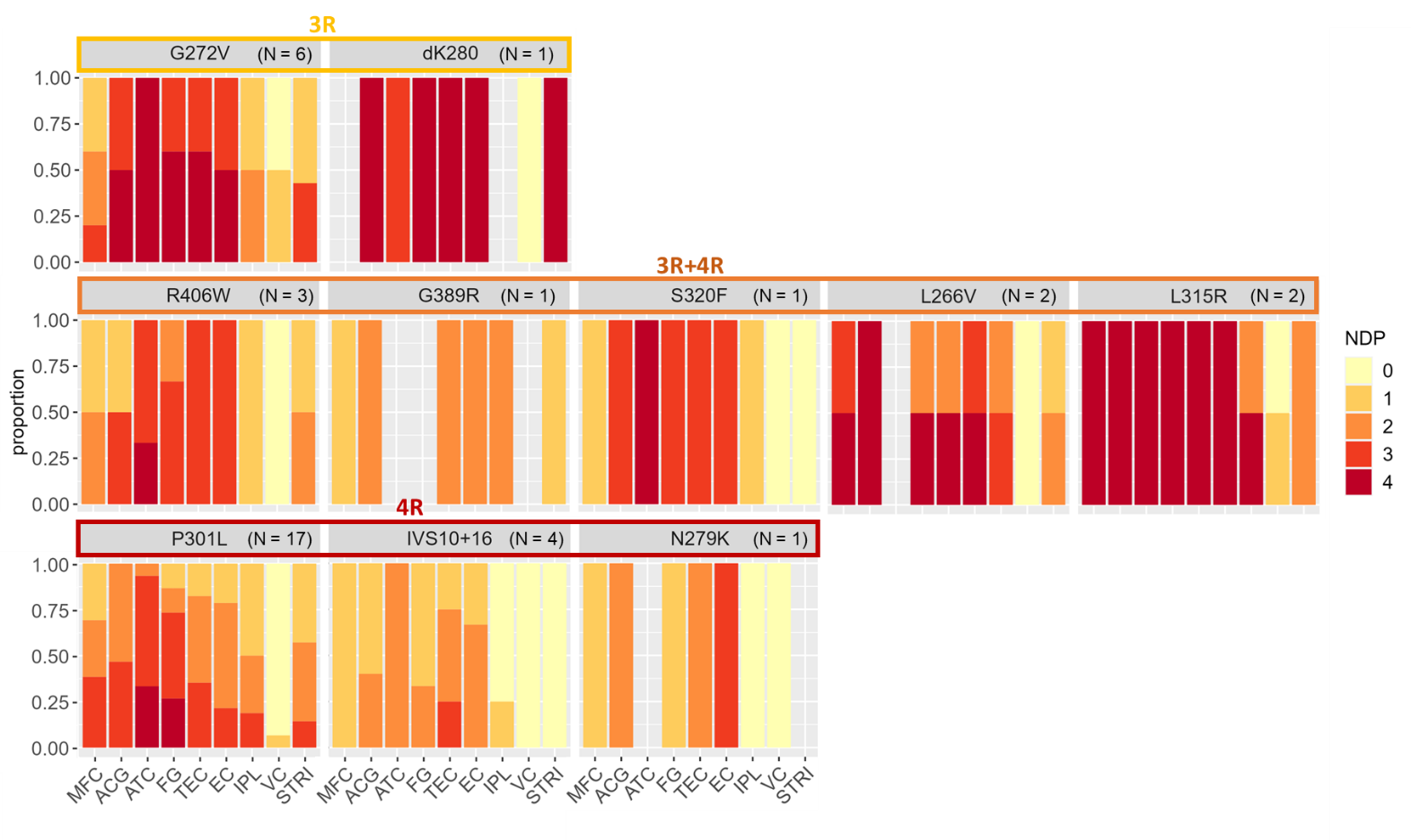
Plots portray the regional distribution of neuronal degeneration (i.e. NDP) in 9 regions with most available data in each *MAPT* variant. We highlight variants belonging to 3R (yellow), 3R+4R (orange) and 4R (red) isoform groups, and the number of cases (N) for each variant. Legend: ACG = anterior cingulate gyrus; ATC = anterior temporal cortex; EC = entorhinal cortex; FG = fusiform gyrus; IPL = inferior parietal lobule; MFC = middle frontal cortex; NDP = neuronal degeneration phase; STRI = striatum; TEC = transentorhinal cortex; VC = visual cortex.

**Supplementary Fig. 13 Fronto-temporal distribution of neuronal degeneration and tau burden in the R406W, IVS10+16 and P301L *MAPT* variants**


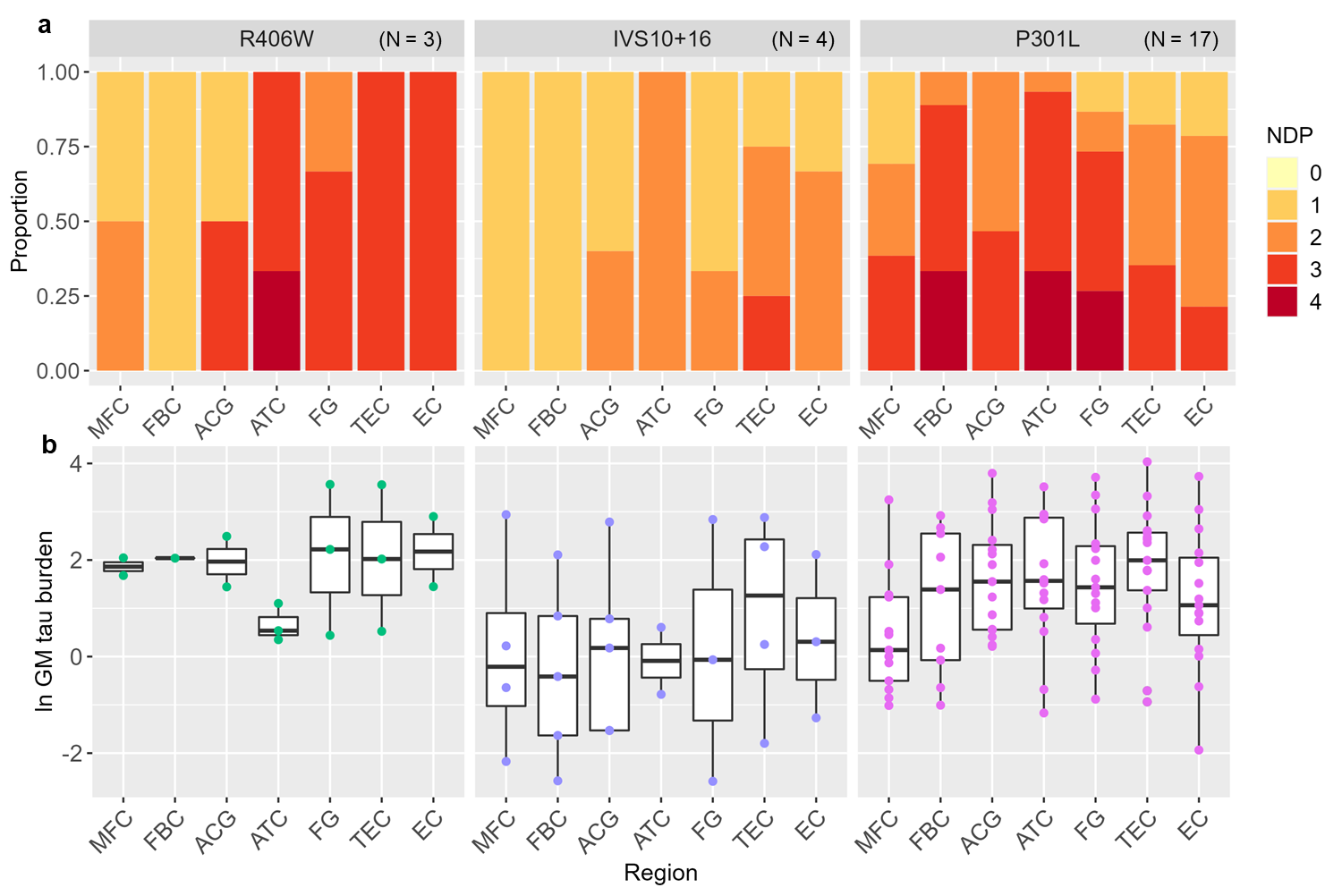


Plots portray frontotemporal patterns of neuronal degeneration (a) and GM tau burden (b) in three specific *MAPT* variants, i.e. R406W (3R+4R), IVS10+16 (4R), P301L (4R). Legend: ACG = anterior cingulate gyrus; ATC = anterior temporal cortex; FBC = frontobasal cortex; GM = grey matter; EC = entorhinal cortex; FG = fusiform gyrus; MFC = middle frontal cortex; TEC = transentorhinal cortex. On the y-axis in panel b, ln GM tau burden indicates the percentage area occupied (%AO) by AT8-positive pixels in GM after natural log transformation.

## TABLES

**Supplementary Table 1 Overview of *MAPT* genetic variants in the cohort and additional genetic information**

| **Case** | **Variant** | **Location** | **Familial relationship** | **Tested gene(s)** | **APOE** |
| --- | --- | --- | --- | --- | --- |
| 1 | c.1820G>T   p.G272V | Exon 9 | G272V family (cases 1-6) | *MAPT* | E3/E3 |
| 2 | c.1820G>T   p.G272V | Exon 9 | G272V family (cases 1-6) | *MAPT* | E3/E4 |
| 3 | c.1820G>T   p.G272V | Exon 9 | G272V family (cases 1-6) | *MAPT* | E3/E4 |
| 4 | c.1820G>T   p.G272V | Exon 9 | G272V family (cases 1-6) | *MAPT* | E4/E4 |
| 5 | c.1820G>T   p.G272V | Exon 9 | G272V family (cases 1-6) | *MAPT* | E3/E3 |
| 6 | c.1820G>T   p.G272V | Exon 9 | G272V family (cases 1-6) | *MAPT* | - |
| 7 | c.1846_1848delAAG p.deltaK280 | Exon 10 | unrelated | *MAPT* | E3/E4 |
| 8 | c.1801C>G p.L266V | Exon 9 |  | *MAPT, C9orf72, GRN* | E3/E4 |
| 9 | c.1801C>G p.L266V | Exon 9 |  | *MAPT, C9orf72* | E3/E3 |
| 10 | c.1949T>G p.L315R | Exon 11 | L315R family (cases 10-11) | *MAPT* | E3/E3 |
| 11 | c.1949T>G p.L315R | Exon 11 | L315R family (cases 10-11) | *None** | - |
| 12 | c.2221C>T p.R406W | Exon 13 | R406W family (cases 12-13) | *MAPT* | E3/E3 |
| 13 | c.2221C>T p.R406W | Exon 13 | R406W family (cases 12-13) | *MAPT* | E3/E4 |
| 14 | c.2221C>T p.R406W | Exon 13 | unrelated | *MAPT* | E3/E4 |
| 15 | c.2170G>A p.G389R | Exon 13 |  | *MAPT, C9orf72, GRN, TARDBP* | E3/E3 |
| 16 | c.1964C>T p.S320 | Exon 11 | unrelated | *MAPT* | E3/E3 |
| 17 | c.1907C>T p.P301L | Exon 10 | P301L family (cases 17-20; 22-33) | *MAPT* | E3/E3 |
| 18 | c.1907C>T p.P301L | Exon 10 | P301L family (cases 17-20; 22-33) | *MAPT* | E2/E3 |
| 19 | c.1907C>T p.P301L | Exon 10 | P301L family (cases 17-20; 22-33) | *MAPT* | E2/E2 |
| 20 | c.1907C>T p.P301L | Exon 10 | P301L family (cases 17-20; 22-33) | *MAPT* | E3/E3 |
| 21 | c.1907C>T p.P301L | Exon 10 | unknown | *MAPT* | E3/E3 |
| 22 | c.1907C>T p.P301L | Exon 10 | P301L family (cases 17-20; 22-33) | *MAPT* | E3/E3 |
| 23 | c.1907C>T p.P301L | Exon 10 | P301L family (cases 17-20; 22-33) | *MAPT* | E3/E3 |
| 24 | c.1907C>T p.P301L | Exon 10 |  | *MAPT, C9orf72, GRN* | E3/E3 |
| 25 | c.1907C>T p.P301L | Exon 10 |  | *MAPT, C9orf72, GRN* | E2/E3 |
| 26 | c.1907C>T p.P301L | Exon 10 | P301L family (cases 17-20; 22-33) | *MAPT* | E2/E4 |
| 27 | c.1907C>T p.P301L | Exon 10 | P301L family (cases 17-20; 22-33) | *MAPT* | E2/E3 |
| 28 | c.1907C>T p.P301L | Exon 10 | P301L family (cases 17-20; 22-33) | *MAPT* | E3/E3 |
| 29 | c.1907C>T p.P301L | Exon 10 | P301L family (cases 17-20; 22-33) | *MAPT* | - |
| 30 | c.1907C>T p.P301L | Exon 10 | P301L family (cases 30-31) | *MAPT, C9orf72, GRN* | E3/E3 |
| 31 | c.1907C>T p.P301L | Exon 10 | P301L family (cases 30-31) | *MAPT* | E3/E3 |
| 32 | c.1907C>T p.P301L | Exon 10 | P301L family (cases 17-20; 22-33) | *MAPT* | - |
| 33 | c.1907C>T p.P301L | Exon 10 | P301L family (cases 17-20; 22-33) | *MAPT* | - |
| 34 | c.1920+16C>T IVS10+16 | Intron 10 | IVS10+16 family (cases 34-35) | *MAPT, C9orf72, GRN* | E2/E3 |
| 35 | c.1920+16C>T IVS10+16 | Intron 10 | IVS10+16 family (cases 34-35) | *MAPT* | E2/E3 |
| 36 | c.1920+16C>T IVS10+16 | Intron 10 |  | *MAPT, C9orf72, GRN, TARDBP, VCP* | E3/E4 |
| 37 | c.1920+16C>T IVS10+16 | Intron 10 |  | *MAPT, C9orf72, GRN, TARDBP, VCP* | E3/E3 |
| 38 | c.1842T>G p.N279K | Exon 10 |  | *MAPT, C9orf72* | E3/E3 |

*Case 11 come from a family known to have the L315R variant and had clinical phenotype and pathological features typical of this mutation, but did not undergo formal genetic testing.

**Supplementary Table 2 Detection algorithms and optical density values in each staining batch**

| **Cohort** | **Staining batch** | **AT8 algorithm (RGB)** | **Hematoxylin algorithm (RGB)** | **OD threshold** |
| --- | --- | --- | --- | --- |
| Erasmus | 1 | 0.351 0.552 0.756 | 0.604 0.663 0.440 | 0.33 |
| Erasmus | 2 | 0.370 0.571 0.731 | 0.629 0.665 0.401 | 0.25 |
| Erasmus | 3 | 0.340 0.573 0.744 | 0.647 0.650 0.398 | 0.37 |
| Penn | 1 | 0.391 0.542 0.743 | 0.646 0.661 0.381 | 0.37 |
| Penn | 2 | 0.322 0.553 0.766 | 0.621 0.664 0.415 | 0.23 |
| Penn | 3 | 0.314 0.574 0.750 | 0.628 0.674 0.387 | 0.29 |

Legend: OD = optical density.

Table displays detection algorithms for the main stain (AT8) and the counterstain (hematoxylin) empirically derived in each staining batch for the two cohorts, and OD values optimized to minimize bias between staining batches based on a set of slides stained in duplicate.

**Supplementary Table 3 Overview of available tissue for %AO measurements per region in each *MAPT* variant and isoform group**

| **Region** | **G272V** | **dK280** | **3R Isoform** | **L266V** | **L315R** | **R406W** | **G389R** | **S320F** | **3R+4R Isoform** | **P301L** | **IVS10+16** | **N279K** | **4R Isoform** | **Tot cohort** |
| --- | --- | --- | --- | --- | --- | --- | --- | --- | --- | --- | --- | --- | --- | --- |
| ACG | 6 | 1 | 7 | 2 | 2 | 2 | 1 | 1 | 8 | 15 | 5 | 1 | 21 | 36 |
| EC | 4 | 1 | 5 | 2 | 2 | 2 | 1 | 1 | 8 | 15 | 3 | 1 | 19 | 32 |
| FBC | 1 | 1 | 2 | 2 | 0 | 1 | 1 | 0 | 4 | 9 | 5 | 1 | 15 | 21 |
| FG | 5 | 1 | 6 | 2 | 2 | 3 | 0 | 1 | 8 | 15 | 3 | 1 | 19 | 33 |
| IPL | 6 | 0 | 6 | 2 | 2 | 1 | 1 | 1 | 7 | 16 | 4 | 1 | 21 | 34 |
| MFC | 5 | 0 | 5 | 2 | 2 | 2 | 1 | 1 | 8 | 13 | 4 | 1 | 18 | 31 |
| TEC | 5 | 1 | 6 | 2 | 2 | 3 | 1 | 1 | 9 | 17 | 4 | 1 | 22 | 37 |
| STG | 0 | 0 | 0 | 2 | 0 | 0 | 1 | 0 | 3 | 5 | 5 | 1 | 11 | 14 |
| STRI | 6 | 1 | 7 | 2 | 2 | 2 | 1 | 1 | 8 | 12 | 2 | 0 | 14 | 29 |
| ATC | 6 | 1 | 7 | 0 | 2 | 3 | 0 | 1 | 6 | 15 | 2 | 0 | 17 | 30 |
| VC | 6 | 1 | 7 | 2 | 2 | 2 | 0 | 1 | 7 | 16 | 4 | 1 | 21 | 35 |
| tot | 50 | 8 | 58 | 20 | 18 | 21 | 8 | 9 | 76 | 148 | 41 | 9 | 198 | 332 |

Legend: ACG = anterior cingulate gyrus; ATC = anterior temporal cortex; EC = entorhinal cortex; FBC = frontobasal cortex; FG = fusiform gyrus; IPL = inferior parietal lobe; MFC = middle frontal cortex; STG = superior temporal gyrus; STRI = striatum; TEC = transentorhinal cortex; VC = visual cortex.

**Supplementary Table 4 Clinical characterization of the cohort and onset year of clinical features**

| **Case** | **Gender** | **Age onset** | **Age death** | **Duration** | **Diagnosis** | **N Visits** | **Word-finding/naming** | **Nonfluent speech** | **Agrammatism** | **Dysarthria** | **Sentence/NOS compr** | **Word/object knowledge** | **Semantic paraphasias** | **Phonemic paraphasias** | **Mute** | **Disinhibition** | **Hypersexuality** | **Apathy / Inertia** | **Empathy** | **Ritualistic behav** | **Hyperorality** | **Parkinsonism** | **Memory/Orientation** |
| --- | --- | --- | --- | --- | --- | --- | --- | --- | --- | --- | --- | --- | --- | --- | --- | --- | --- | --- | --- | --- | --- | --- | --- |
| 1 | F | 45 | 54 | 9 | bvFTD | 3 |  |  | 2 |  |  |  | 2 | 2 |  | 0 | 0 | 0 | 0 | 0 | 0 |  | 2 |
| 2 | F | 47 | 67 | 20 | bvFTD | 1 | 7 |  |  |  | 12 |  |  |  | 7 | 0 |  | 7 | 3 | 3 | 3 | 7 | 7 |
| 3 | F | 47 | 54 | 7 | bvFTD | 2 |  |  |  |  |  | 5 |  | 6 |  | 5 |  | 0 | 0 |  |  |  |  |
| 4 | M | 41 | 49 | 8 | bvFTD | 4 |  |  |  |  |  |  |  |  |  | 0 |  | 0 | 0 | 0 | 0 |  | 3 |
| 5 | M | 42 | 51 | 9 | bvFTD | 2 | 2 |  |  |  |  |  |  |  |  | 0 |  | 0 | 2 | 0 | 0 |  |  |
| 6 | M | 42 | 49 | 7 | bvFTD | 6 | 0 |  |  |  |  | 1 |  |  |  | 1 |  |  | 1 | 1 |  |  | 1 |
| 7 | F | 52 | 63 | 11 | bvFTD | 8 |  |  |  |  |  | 2 |  |  | 4 | 0 |  | 0 | 2 | 0 | 2 |  | 2 |
| 8 | F | 31 | 34 | 3 | bvFTD | 3 | 0 | 0 |  |  | 2 |  |  |  |  | 0 |  | 0 |  | 2 |  |  | 0 |
| 9 | F | 24 | 31 | 7 | bvFTD | 2 | 4 | 4 | 4 | 6 |  |  |  |  |  | 6 |  | 0 | 0 | 6 | 0 | 6 |  |
| 10 | F | 54 | 63 | 9 | bvFTD | 4 | 3 |  |  |  |  | 6 |  |  | 7 | 0 | 5 | 3 | 0 | 3 | 0 | 7 | 5 |
| 11 | M | 52 | 57 | 5 | bvFTD | 2 | 0 |  |  |  |  |  |  | 0 | 7 | 0 |  | 0 | 0 | 0 | 0 |  |  |
| 12 | M | 50 | 70 | 20 | bvFTD | 1 |  |  |  |  |  |  |  |  |  | 3 | 0 | 9 |  | 0 | 13 |  | 13 |
| 13 | F | 58 | 71 | 13 | bvFTD | 4 |  |  |  |  |  |  |  |  | 6 | 0 |  | 3 |  | 0 | 3 |  | 4 |
| 14 | F | 58 | 75 | 17 | bvFTD | 8 | 9 |  |  |  | 9 |  |  |  | 10 | 0 | 10 | 13 |  | 0 |  | 14 |  |
| 15 | F | 40 | 43 | 3 | bvFTD | 1 |  | 3 |  | 0 |  |  |  |  | 3 | 0 | 0 | 0 |  |  | 0 |  |  |
| 16 | M | 38 | 53 | 15 | bvFTD | 11 | 0 |  |  |  | 10 | 11 |  |  |  | 0 |  | 9 | 0 | 9 | 9 |  | 9 |
| 17 | F | 54 | 66 | 12 | bvFTD | 1 |  |  |  |  | 3 |  |  |  | 10 | 0 |  | 0 | 3 | 3 | 4 | 10* | 7 |
| 18 | M | 45 | 52 | 7 | bvFTD | 8 | 1 |  |  |  | 1 | 1 |  |  | 3 | 1 |  | 2 |  |  |  |  |  |
| 19 | F | 56 | 76 | 20 | bvFTD | 2 |  |  |  |  |  |  |  |  | 19 | 0 |  | 12 | 3 | 3 | 3 | 15 |  |
| 20 | M | 58 | 66 | 8 | bvFTD | 3 | 0 |  |  | 3 | 1 |  | 2 |  |  | 0 |  |  | 3 | 2 |  |  | 2 |
| 21 | M | 39 | 46 | 7 | bvFTD | 1 |  |  |  |  |  |  |  |  |  | 0 |  | 0 |  | 0 |  |  | 0 |
| 22 | F | 56 | 66 | 10 | bvFTD | 5 | 0 |  |  |  |  | 2 | 2 | 2 | 6 | 0 | 6 | 0 | 0 | 0 | 0 |  |  |
| 23 | M | 49 | 52 | 3 | bvFTD | 3 | 0 |  |  |  | 3 | 2 | 0 |  |  | 2 |  | 0 | 0 | 0 |  |  |  |
| 24 | F | 51 | 71 | 20 | FTD-NOS^¥^ | 0 |  |  |  |  |  |  |  |  |  |  |  |  |  |  |  |  |  |
| 25 | F | 56 | 65 | 9 | bvFTD | 2 | 4 |  |  | 4 | 4 |  |  | 4 | 9 | 0 |  | 9 |  | 0 |  | 4 | 4 |
| 26 | M | 51 | 60 | 9 | bvFTD | 12 | 0 |  |  |  | 2 | 0 | 0 |  | 5 | 4 |  | 0 | 3 | 1 |  | 5 | 1 |
| 27 | F | 50 | 64 | 14 | bvFTD | 1 | 0 |  |  |  |  |  |  |  | 13 | 0 |  | 0 | 0 |  | 0 | 12 |  |
| 28 | M | 52 | 64 | 12 | bvFTD | 5 | 0 |  |  |  | 4 | 4 |  | 4 |  | 0 |  | 4 | 4 | 4 | 4 | 9* | 4 |
| 29 | M | 53 | 55 | 2 | bvFTD | 1 | 0 |  |  |  |  |  |  |  |  | 0 |  | 0 |  |  | 0 |  |  |
| 30 | F | 53 | 64 | 11 | bvFTD^#^ | 0 |  |  |  |  |  |  |  |  |  |  |  |  |  |  |  |  |  |
| 31 | M | 57 | 68 | 11 | bvFTD^#^ | 0 |  |  |  |  |  |  |  |  |  |  |  |  |  |  |  |  |  |
| 32 | M | 53 | 57 | 4 | bvFTD | 5 | 2 |  |  |  | 4 | 2 |  | 2 |  | 0 |  | 0 | 0 | 1 | 0 | 2 | 2 |
| 33 | M | 57 | 65 | 8 | bvFTD | 4 | 0 |  |  |  |  |  |  |  |  | 0 |  | 0 | 0 | 0 | 0 |  |  |
| 34 | M | 46 | 48 | 2 | PSP^&^ | 0 |  |  |  |  |  |  |  |  |  |  |  |  |  |  |  | 0 |  |
| 35 | F | 55 | 62 | 7 | bvFTD | 1 |  |  |  |  |  |  |  |  |  | 0 |  | 0 |  | 0 | 0 | 5 |  |
| 36 | M | 60 | 68 | 8 | bvFTD | 3 | 4 |  |  |  |  |  |  |  |  | 0 |  |  | 0 | 0 | 0 |  |  |
| 37 | F | 54 | 64 | 10 | bvFTD | 7 | 10 |  |  |  |  | 10 |  | 12 |  | 0 | 0 | 0 | 0 | 0 | 0 | 10 |  |
| 38 | F | n/a | 49 | n/a | FTD-NOS^¥^ | 0 |  |  |  |  |  |  |  |  |  |  |  |  |  |  |  |  |  |

bvFTD = behavioral variant frontotemporal dementia; FTD-NOS = frontotemporal dementia not otherwise specified; n/a = not available; PSP = progressive supranuclear palsy.

Table reports onset year of each clinical feature. A zero (= 0) indicates that a certain clinical feature is present from disease onset. Absent values indicate that a certain clinical feature has not been reported in the clinical reports.

^#^No clinical reports available, but a bvFTD diagnosis was indicated in the autopsy report
^&^No clinical reports available, but a PSP diagnosis was indicated in the autopsy report
^¥^No clinical reports available and no conclusive information on clinical diagnosis from the autopsy report
*No ascertained rigidity or bradykinesia due to a lack of formal neurological examinations at follow-up, but gait impairment suggestive of parkinsonism

**Supplementary Methods**

*Model 1: Relation between neuronal degeneration and grey matter tau burden*

This linear mixed-effect model used ln-transformed GM %AO (i.e. GM tau burden) as dependent variable, cases as random effects, neuronal degeneration phase (NDP) scores as fixed-effect main predictor and region as fixed-effect covariate. The model was followed by type III ANOVA with Satterthwaite's method to test the association between NDP scores and GM tau burden.

*Model 2: Regional analysis of grey matter tau burden*

We performed this analysis including nine regions with most available data. We used a linear mixed-effect model with ln-transformed GM %AO (i.e. GM tau burden) as dependent variable, cases as random effects, region as fixed-effect main predictor, and NDP and isoform groups as fixed-effect covariate. The model was followed by type III ANOVA with Satterthwaite's method to assess the main effect of region on the model, then least-square means corrected for random effects and covariate from the model were estimated in each region. We repeated this analysis within each isoform group to study differential regional distribution patterns of GM tau burden within each group.

*Model 3: Regional analysis of neuronal degeneration severity*

We performed this analysis including nine regions with most available data. Using a cumulative linked mixed-model, we assessed the effect of region as fixed-effect main predictor on NDP scores (i.e. 0-4) as dependent variable, with cases as random effects and isoform group as fixed-effect covariate. We used a Likelihood Ratio Test comparing this model to a nested model (without region) to assess the significance of region as main predictor. We repeated this analysis within each isoform group to assess the association of region with neuronal degeneration severity within each group.

*Model 4: Comparison of grey matter tau burden between isoform groups irrespective of brain region*

This linear mixed-effect model used ln-transformed GM %AO (i.e. ln GM tau burden) as dependent variable, cases as random effects, isoform group as fixed-effect main predictor, and region as fixed-effect covariate. The model was followed by type III ANOVA with Satterthwaite's method and by pairwise *post-hoc* comparisons.

*Model 5: Frequency of very severe neuronal degeneration in different isoform groups irrespective of brain region*

We used a cumulative linked mixed-model with neuronal degeneration phase score as dependent variable, cases as random effects, isoform group as fixed-effect main predictor, and region as fixed-effect covariate. We used a Likelihood Ratio Test comparing this model to a nested model (without isoform group) to assess the significance of isoform group as main predictor.

*Model 6: Relative severity of cortical neuronal and glial pathology*

This analysis was performed using a cumulative linked mixed-model with ordinal scores of neuronal/glial tau pathology as dependent variable, cases as random effects, isoform group as fixed-effect main predictor, and region as fixed-effect covariate. A Likelihood Ratio Test comparing the full model to a nested model (without isoform group) was used to assess the significance of isoform group as main predictor.
